# *Piper nigrum* Extract as an Adjuvant in a Collagen System for Infected Wound Healing: Therapeutic Synergy and Biocompatibility

**DOI:** 10.3390/antibiotics14111166

**Published:** 2025-11-17

**Authors:** Virgina Silviana Becherescu Barbu, Ioana Cristina Marinas, Diana Madalina Gaboreanu, Ionela Cristina Voinea, Oana Brincoveanu, Elisabeta-Irina Geana, Ovidiu-Cristian Oprea, Adina Boldeiu, Andra Maria Paun, Catalina Mares, Marian Angheloiu, Alice-Stefania Serbanoiu, Speranta Avram

**Affiliations:** 1 Department of Anatomy, Animal Physiology and Biophysics, Faculty of Biology, University of Bucharest, 050095 Bucharest, Romania; virginia.becherescu@phanos.ro (V.S.B.B.); paun.andra-maria22@s.bio.unibuc.ro (A.M.P.); catalina.mares@bio.unibuc.ro (C.M.); speranta.avram@bio.unibuc.ro (S.A.); 2 Phanos Technology SRL, 235300 Corabia, Romania; 3 The Research Institute of the University of Bucharest (ICUB), 050095 Bucharest, Romania; 4 SC Deltarom SRL—Centre for Research and Innovative Services in Advanced Biotechnology, 1-2 Bucuresti-Giurgiu Street, 087040 Giurgiu, Romania; marian.angheloiu@sanimed.ro; 5 Department of Botany and Microbiology, Faculty of Biology, University of Bucharest, Splaiul Independentei 91-95, 050095 Bucharest, Romania; gaboreanu.diana-madalina@s.bio.unibuc.ro (D.M.G.); alice.serbanoiu@s.unibuc.ro (A.-S.S.); 6 Department of Biochemistry and Molecular Biology, Faculty of Biology, University of Bucharest, 91-95 Splaiul Independentei, 050095 Bucharest, Romania; ionela-cristina.voinea@bio.unibuc.ro; 7 National Institute for Research and Development in Microtechnologies (IMT-Bucharest), 077190 Bucharest, Romania; oana.brincoveanu@imt.ro (O.B.); adina.boldeiu@imt.ro (A.B.); 8 National Research & Development Institute for Cryogenic and Isotopic Technologies-Ramnicu Valcea Romania, Strada Uzinei, No. 4, 240050 Râmnicu Vâlcea, Romania; irina.geana@icsi.ro; 9 National Centre for Micro- and Nanomaterials, National University of Science and Technology Politehnica Bucharest, 060042 Bucharest, Romania; ovidiu.oprea@upb.ro; 10 Academy of Romanian Scientists, 3 Ilfov St., 050045 Bucharest, Romania; 11 Department of Inorganic Chemistry, Physical Chemistry and Electrochemistry, Faculty of Chemical Engineering and Biotechnologies, National University of Science and Technology Politehnica of Bucharest, 011061 Bucharest, Romania

**Keywords:** *P. nigrum* extract, collagen–arginine composite, antimicrobial activity, antioxidant activity, synergism, cefazoline, biocompatibility

## Abstract

**Background/Objectives**: Natural plant-based compounds, especially black pepper extract, are known to have anti-inflammatory, antibacterial, and antioxidant qualities that promote procollagen formation and wound healing. This study focused on developing a collagen-based composite enriched with *P. nigrum* extract in powder form, designed to enhance the efficacy of the antibiotic cefazolin while promoting the healing of chronic wounds. **Methods**: The polyphenolic *P. nigrum* extract was obtained by ultrasound-assisted extraction and was characterised by UHPLC-MS/MS and spectrophotometry. Antimicrobial and antioxidant activities were assessed using conventional methods. Pharmacokinetic and pharmacodynamic parameters were evaluated for the specific taxon compounds using Deep-RK. *P. nigrum* extract was incorporated into a collagen hydrogel with arginine and freeze-dried. The powders were characterised by FTIR, SEM, TGA-DSC, and DLS. The antimicrobial activity and potential synergistic effects with cefazolin were evaluated on reference microbial strains and isolates from infected wounds. Biocompatibility and hemocompatibility were evaluated, as well as wound closure in vitro. **Results**: Polyphenols, including phenolic acids, stilbenes, anthocyanins, and flavonoids, which provide a potent antioxidant capacity through electron transfer mechanisms (FRAP, CUPRAC), were abundant in the *P. nigrum* extract. FTIR and SEM analyses confirmed the integration of phenolic compounds into the collagen–arginine matrix without protein denaturation. TGA–DSC data showed thermal stabilisation at moderate extract concentrations. The extract exhibited predominantly bacteriostatic antibacterial activity and antibiofilm effects, with synergy/additivity with cefazolin, especially at medium doses. Tests on keratinocytes confirmed biocompatibility, and hemocompatibility demonstrated an excellent safety profile, with protection against AAPH-induced oxidative stress. **Conclusions**: Overall, collagen powders with *P. nigrum* extract at moderate/low concentrations combine stability, antibiotic-enhanced activity, and cellular compatibility, making them promising adjuvants for the topical treatment of chronically infected wounds.

## 1. Introduction

The complex biological process of wound healing involves the simultaneous and sequential occurrence of haemostasis, inflammation, proliferation, and remodelling [1]. Hard-to-heal lesions, such as diabetic foot ulcers or venous leg ulcers, disrupt these sequences due to oxidative stress, poor vascularisation, persistent inflammation, and infection, slowing recovery and raising medical costs [2]. Natural compounds with antioxidant, antibacterial and anti-inflammatory properties that stimulate procollagen production, especially natural compounds derived from plants, have demonstrated an increasing ability to promote wound healing [3].

In this context, the development of functionalised materials that actively contribute to the intensification of re-epithelisation processes is a promising strategy. Several studies have shown that black pepper extract, which has a rich profile of polyphenols and alkaloids, including piperine, has antibacterial, anti-inflammatory, and antioxidant properties [4,5], with the ability to reduce microbial load and protect the extracellular matrix from free radical-induced degradation. Collagen, an essential constituent of connective tissue, facilitates cell migration and proliferation and provides a biomimetic structure that facilitates the formation of granulation tissue and re-epithelialisation [6]. Nitric oxide, a therapeutic precursor of arginine, stimulates angiogenesis, vasodilation, and tissue repair. For this reason, it stabilises the powder formulation when added to the collagen composition [7,8,9].

Formulating these components as powders offers several advantages over traditional dressings, including easy and flexible application to irregular surfaces [10], controlled and sustained release of bioactive compounds, acting as a support for various active principles [10,11], and improved long-term stability [12]. A functionalised powder, including collagen, arginine, and *P. nigrum* extract, may provide a pro-regenerative, antibacterial, and antioxidant approach to accelerating the healing of infected wounds.

Collagen-based dressings are widely utilised in clinical and biomedical settings because of their biocompatibility, minimal immunogenicity, and ability to duplicate the extracellular matrix (ECM), which stimulates cell adhesion and proliferation [3]. Native collagen’s capacity to cure infected or oxidatively stressed wounds is restricted when it lacks innate antibacterial or antioxidant capabilities [13].

Recent studies indicated that the *P. nigrum* extract appeared to be a suitable alternative [3,10,11,12,13,14]. The hydroalcoholic extract of *P. nigrum*, which is rich in polyphenols and alkaloids, is mostly composed of phenolic acids and piperine. Furthermore, natural compounds such as flavonoids and stilbenes have distinct therapeutic properties [14]. Its adaptability explains its pro-regenerative, antibacterial, and antioxidant (SET/HAT) qualities, making it useful for topical therapies and biomaterial formulations. This is associated with reduced levels of inflammatory cytokines and increased collagen deposition [15]. The *P. nigrum* extract exhibits antimicrobial activity against numerous strains, both foodborne and clinical: *Staphylococcus aureus*, *Streptococcus mutans*, *Enterococcus faecalis*, *Salmonella* sp., *Proteus mirabilis*, *Pseudomonas aeruginosa*, *Candida albicans*, *Escherichia coli*, *Klebsiella pneumoniae*, *T. mentagrophytes*, *T. rubrum*, *Aspergillus niger* [5,16,17,18,19,20]. Hydrogels containing piperine, a particular constituent of the *P. nigrum* hydroalcoholic extract, have shown considerably quicker wound healing in mouse models in in vivo tests, much like carbopol–Aloe vera formulations. This is related to lower levels of inflammatory cytokines and higher collagen deposition [3]. Black pepper’s bioactive metabolite, piperonylic acid, activates the EGFR pathway, increases the production of type I collagen, and modifies the cytokines IL-6, IL-1β, and TNF-α in mice. *P. nigrum* essential oil (EO) has shown promise in increasing collagen production, modifying the extracellular matrix, and regulating cutaneous fibroblast function. Recent research indicates that the addition of black pepper hydroalcoholic extracts, or EOs, to polymer networks boosts their antibacterial activity and stimulates the production of more collagen by fibroblasts [21]. Collagen-based hydrogels have demonstrated long-term benefits in tissue repair owing to their superior biocompatibility and resemblance to the extracellular matrix architecture [22,23]. Combining structural support and collagen architecture compatible with eukaryotic cells with the bioactivity of specific compounds from the *P. nigrum* extract can lead to the development of a multifunctional dressing capable of protecting the wound from oxidative stress and microbial invasion, promoting the proliferation of fibroblasts or keratinocytes, and supporting collagen deposition and re-epithelialisation. In this context, the aim of the study was to develop and characterise a collagen-based composite enriched with a *P. nigrum* ethanolic extract, formulated as a powder with antioxidant potential and the ability to improve the activity of the cefazolin. Cefazolin is a well-known antibiotic for treating wound infections due to its safety and ability to penetrate skin tissue [24,25,26,27,28]. It can be used as an adjuvant alongside other techniques as well as a first-line therapy for Gram-positive infections. Its usage in chronic wounds must be tailored to the bacteriological profile because of its low efficacy against Gram-negative bacteria [29]. In this context, functionalised collagen powder was tested in the presence of cefazolin to highlight the interaction between the antibiotic and the active principles from the *P. nigrum* extract.

As a result, the purpose of this study is to create and characterise a collagen-based composite powder functionalised with an ethanolic extract of *P. nigrum* and cefazolin, with the hypothesis that the combination improves the material’s antioxidant, antibacterial, and wound-healing properties via synergistic or additive effects between the natural bioactive compounds and the antibiotic.

In the field of structural bioinformatics, fragment-based modelling methods and structural segment libraries are used to predict the molecular characteristics associated with functional compounds. With expertise in the development of new therapeutic agents and the evaluation of the pharmacokinetic and pharmacodynamic parameters of natural compounds, this bioinformatic study was conducted to explore the underlying mechanisms of their therapeutic effects [30,31,32,33]. Thus, certain phytocompounds from *P. nigrum* were examined utilising in silico approaches, with bioinformatics tools used to evaluate their characteristics comprehensively. The SwissADME platform (https://www.swissadme.ch/), DeepPK (https://biosig.lab.uq.edu.au/deeppk/), and other data support resources were used in computational analyses to predict physicochemical properties, drug-likeness, ADME-Tox profiles (absorption, distribution, metabolism, excretion, and toxicity), and pharmacodynamic behaviour [34].

## 2. Results

### 2.1. Insights into the Phytochemistry and Therapeutic Potential of P. nigrum Hydroalcoholic Extract

#### 2.1.1. Physico-Chemical Characterisation of *P. nigrum* Extract

Polyphenols are bioactive compounds with an important antioxidant role. Table 1 shows the results for the total polyphenol and flavonoid content. The extraction yield was 4.06%, a value obtained by comparing the plant material and the freeze-dried extract obtained, a value much lower than that reported by Luca et al. [35].

The moderate value suggests the presence of phenolic compounds in the *P. nigrum* extract, which could contribute to its ability to neutralise free radicals and offer protection against oxidative stress. The results were expressed as µg GAE/mL of extract to correlate with the extract concentration included in the formulations. The crude extract obtained has a polyphenol content of 497.57 ± 4.81 µg GAE/mL and a flavonoid content of 80.25 ± 1.40 µg QE/mL.

A subgroup of polyphenols, flavonoids, has anti-inflammatory and cell-protective properties. The figure, 16.13% of the TPC, suggests that other phenolic compounds—such as phenolic acids, aromatic amino acids, coumarins, lignans, stilbenes, etc.—rather than flavonoids constitute the extract’s main polyphenols. However, the presence of flavonoids supports the diversity of bioactive compounds in the extract.

**Figure 1:**
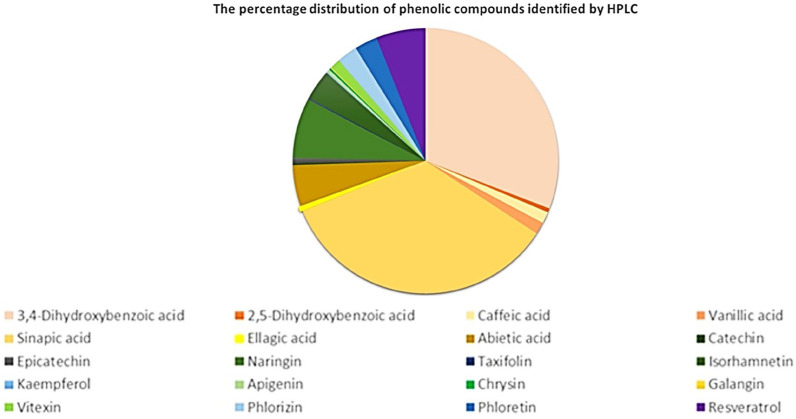
Percentage distribution of phenolic compounds identified by HPLC: phenolic acids (yellow–orange–brown), flavonoids (green), dihydrochalcones (blue) and others (purple).

The hydroalcoholic extract of *P. nigrum* seeds contained phenolic acids such as sinapic acid (2365.64 µg/L), 3,4-dihydroxybenzoic acid (2107.18 µg/L), abietic acid (343.01 µg/L), caffeic acid (96.97 µg/L), and vanillic acid (98.75 µg/L), as well as naringin (498.81 µg/L), isohamnetin (253.61 µg/L), phlorhizin (170.87 µg/L), and phloretin (189.21 µg/L) were the most abundant flavonoids. The results obtained by HPLC showed that the percentage of individual compounds identified represents 1.74% of the total polyphenols determined by the Folin–Ciocalteu method and 1.66% of the total flavonoids determined by the AlCl_3_ method. The percentage distribution of phenolic acids, flavonoids, dihydrochalcones and resveratrol quantified by UHPLC is presented in Figure 1. The percentage distribution of phenolic compounds identified by HPLC showed the predominance of 3,4-dihydroxybenzoic acid and sinapic acid, while other phenolic compounds, such as isorhamnetin and naringin, were present in smaller proportions.

Chemical compounds such as phenolic acids, flavonoids, isoflavones, stilbenes, and anthocyanidin derivatives were able to be identified and quantified in the *P. nigrum* seed extract. Table 2 presents the molecular formula and retention time of the main compounds identified based on the mass-to-charge ratio (*m*/*z*) and the MS/MS fragment formed during negative-mode ESI. Most of the identified compounds exhibited antitumor, antimicrobial, and anti-inflammatory properties [36,37,38,39,40], as well as re-epithelialisation properties [41,42].

UHPLC-ESI-HRMS investigations were used to characterise the components in the *P. nigrum* seeds extract. The total ion current (TIC) chromatogram in positive ion mode, covering a scan range of 69 to 370 *m*/*z*, enabled the identification of many taxon-specific metabolites. Table 2 summarises the retention times, precursor ion masses, and associated fragment ions for each compound. Among the identified substances are piperine-type alkaloids and lactam derivatives (Piperolactam A and D), polycyclic compounds such as Cenpharadione A, saturated and unsaturated fatty acids (Tetracosanoic acid, 2-Butenedioic acid), as well as phenolic acids, such as 3,4-Methylenedioxycinnamic acid. These compounds have been identified in numerous studies [38,39,40,41] and support a beneficial multimodal profile in controlling inflammation [43] and infection [44,45,46], reducing oxidative stress [44] and supporting tissue repair [47], all essential aspects in the management of chronic wounds.

#### 2.1.2. Antioxidant Activity

The DPPH test measures the extract’s ability to neutralise free radicals. A lower IC_50_ value suggests a higher antioxidant activity. The DPPH radical scavenging ability was moderate (IC_50_ = 41.14 ± 4.33 µL/mL), indicating good antiradical activity, although lower than pure antioxidants (Trolox). The dose-DPPH radical scavenger curves can be found in the Supplementary Materials, Figure S1. In contrast, the values obtained through the CUPRAC (1.04 ± 0.03 mM ET/mL) and FRAP (2.15 ± 0.02 mM ET/mL) tests indicate considerable reducing power, which confirms the presence of compounds capable of donating electrons and stabilising reactive species through electron transfer mechanisms. The fact that the FRAP value almost doubles the CUPRAC result suggests that the extract’s predominant mechanism is the reduction of iron ions, which correlates with previous data showing that *P. nigrum* extracts exhibit strong redox capacity in Fe^3+^-dependent systems [48]. To our knowledge, the evaluation of reducing capacity using the CUPRAC method for the *P. nigrum* extract was performed for the first time.

The *P. nigrum* extract had a higher reducing capacity (FRAP) but also significant radical scavenging activity (TEAC). For *P. nigrum*, values between 0.057–1.24 mM ET/g of plant material have been reported, but the extraction methods and solvents used differ, which may account for the variability observed in the reported values [35,49,50]. Therefore, the value of 185.76 ± 9.77 µM ET/g freeze-dried extract (7.54 ± 0.40 µM ET/g dry plant material) was consistent with data from the specialised literature; the differences could be attributed to the extraction method and possibly the specific phytochemical composition of the batch of raw material used [50,51].

According to the FRAP, CUPRAC, and DPPH tests, the extract of *P. nigrum* seeds possesses antioxidant qualities due to its content of phenolic compounds. These findings suggest that it might be used as a natural agent to protect against oxidative damage.

#### 2.1.3. Antimicrobial Activity of *P. nigrum* Extract

Patients with chronic wounds have a lower quality of life, higher morbidity, and higher treatment costs, which represent a significant public health problem [52]. One of the main causes of persistent lesions and delayed healing is bacterial colonisation and infection, especially in the form of microbial biofilms [53]. By forming biofilms, bacteria significantly increase their resistance to drugs and the host’s immune system, rendering traditional treatment ineffective [54]. The antibacterial and antibiofilm properties of the active principles used for this purpose are crucial, as they can provide adjuvant strategies for infection control and promote tissue regeneration [44,45]. The use of both clinical and reference (e.g., ATCC) strains provides a balanced approach, allowing evaluation of the compounds’ efficacy under realistic infection conditions while maintaining experimental reliability through well-characterised reference strains.

The *P. nigrum* extract displayed strain-dependent antibacterial effects. MIC values were 62.5 µL/mL for *P. aeruginosa* ATCC 27853 and *K. pneumoniae* B1K, and 125 µL/mL for *P. mirabilis* ATCC 2924S, *E. coli* ATCC 25922, *P. aeruginosa* 1014, and *Staphylococcus* spp. (including MRSA). By contrast, the ethanol control required ≥250 µL/mL, indicating that the activity observed was attributable to the extract rather than the solvent. The extract did not show microbicidal effects, the MMC values being more than 500 µL/mL (Table 3), consistent with a primarily bacteriostatic profile.

A biofilm is a community of bacteria attached to a surface, surrounded by a protective matrix of polysaccharides [55]. In infected wounds (e.g., diabetic ulcers, pressure sores, surgical wounds), bacteria do not just remain in a free-floating (planktonic) form, but they also form adherent biofilms on the tissue [56,57]. These biofilms create a protective barrier that reduces the effectiveness of antibiotics, shields bacteria from the immune response, maintains inflammation, and delays healing [58,59]. It is estimated that over 60–80% of chronic wounds have bacterial biofilms [53,60].

According to Table 3, it can be observed that the *P. nigrum* extract inhibited the adherence of *S. aureus* ATCC 25923 cells (MBEC = 62.5 µL/mL) at a moderate concentration, while for *MRSA* cells, the adherence inhibition was achieved at a value of 31.25 µL/mL, proving that the cells of this methicillin-resistant strain were more sensitive to the extract. In the case of Gram-negative bacteria, *P. aeruginosa* 1014, *K. pneumoniae* B1K, and *P. mirabilis* 11P cells, strains isolated from infected wounds, showed a lower anti-biofilm effect compared to a similar concentration of solvent in the extract (starting from a 50% ethanol stock solution). In contrast, other strains (e.g., *E. faecalis*, *E. coli*) have a higher MBEC similar to the solvent used (125–250 µL/mL), suggesting that the effect was due to ethanol and not necessarily the extract.

Therefore, the *P. nigrum* extract showed a promising biofilm eradication effect on some important pathogenic bacteria (*MRSA*, *Pseudomonas*, *Klebsiella*, *Proteus*), some isolated from infected wounds. Biofilms are one of the biggest problems in chronic and medical device-associated infections.

The data suggests that *P. nigrum* extract has potential as an adjuvant agent in the treatment of infected wounds by inhibiting and eradicating bacterial biofilms (particularly MRSA, *P. aeruginosa*, *K. pneumoniae*), reducing microbial resistance, and facilitating healing by limiting the persistence of chronic infection.

**Figure 2:**
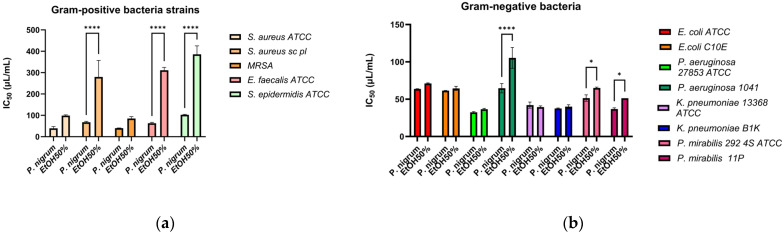
The antibacterial activity against Gram-positive (**a**) and Gram-negative (**b**) strains is represented as IC_50_ values, which are related to the activity of the solvent control. Statistical analysis is performed in triplicate using the two-way ANOVA method (Sidak’s multiple comparisons test), and significance is expressed as follows: * *p* < 0.05, **** *p* < 0.0001.

IC_50_ analysis makes it possible to compare the extract’s and solvent’s efficacy. The extract has a higher antibacterial action when its IC_50_ is lower, and smaller doses are needed to achieve 50% suppression of microbial viability. In Figure 2a the IC_50_ values against Gram-positive bacteria are represented. The *P. nigrum* extract has significantly lower IC_50_ values compared to ethanol for all strains, confirming that the antimicrobial effect was real and not due to the solvent. However, statistically significant results were only observed for the *S. aureus* sc pl, *E. faecalis*, and *S. epidermidis* strains (*p* < 0.0001). *P. nigrum* extract has antimicrobial activity against clinically relevant Gram-positive bacteria (*S. aureus*, *E. faecalis*, and *S. epidermidis*). The results support the extract’s potential as a complementary agent for skin infections caused by Gram-positive bacteria. The dose–response growth curves can be found in the Supplementary Materials Figure S2a–m.

For nearly all Gram-negative strains (Figure 2b), the *P. nigrum* extract demonstrated lower IC_50_ values than the solvent, reflecting its enhanced ability to inhibit bacterial growth. Only for the *P. aeruginosa* 1014 (*p* < 0.0001), *P. mirabilis* 292 4S ATCC, and *P. mirabilis* 11 (*p* < 0.05) strains, the differences were statistically significant. The *P. nigrum* extract demonstrated antimicrobial activity against Gram-negative bacteria as well, although these bacteria are normally more resistant due to their extracellular membrane composed of lipopolysaccharides [61].

### 2.2. Bioinformatics Approach to Natural Compounds from P. nigrum Extract

As part of the bioinformatics analysis, a selection of phytochemical compounds extracted from *P. nigrum* was investigated. These compounds were chosen due to their limited exploration in the existing literature, particularly regarding their structural characteristics and pharmacokinetic/pharmacodynamic (PK/PD) profiles. Despite the extensive traditional use of *P. nigrum*, many of its bioactive constituents remain poorly characterised from a pharmacological and computational perspective. Therefore, this study aimed to bridge this knowledge gap through a comprehensive in silico approach. To assess the drug-likeness and therapeutic potential of the selected compounds, several molecular descriptors critical to early-stage drug discovery were analysed. These include Lipinski’s Rule of Five—commonly used to evaluate oral bioavailability based on molecular weight, lipophilicity (logP), hydrogen bond donors, and hydrogen bond acceptors—and the Veber rule, which considers rotatable bonds and polar surface area as predictors of oral bioavailability. In addition to these filters, the bioavailability score was calculated to estimate the likelihood of a compound exhibiting sufficient systemic exposure when administered orally.

According to the data obtained from the SwissADME database (Table 4 and Table 5), all eight analysed compounds demonstrated favourable bioavailability scores. Specifically, compounds 1 through 4 exhibited a bioavailability score of 0.55, while compounds 5 through 8 showed a higher score of 0.85, indicating good potential for oral bioavailability. Seven out of the eight compounds fully comply with Lipinski’s Rule of Five, a widely accepted guideline for evaluating drug-likeness. These compounds exhibit a molecular weight (MW) of less than or equal to 500 Da, contain fewer than 5 hydrogen bond donors, fewer than 10 hydrogen bond acceptors, and have a logP value below 5. The one exception—Tetracosanoic acid—exceeds the threshold for one or more of these parameters, likely due to its long aliphatic chain. Furthermore, all compounds display Topological Polar Surface Area (TPSA) values in the range of 37 Å^2^ to 72 Å^2^. These values fall within the optimal range for oral absorption, further supporting the compounds’ potential as orally bioavailable agents. In terms of lipophilicity, the LOGP values range from 0.32 to 5.62, indicating moderate lipophilicity, which is generally associated with favourable membrane permeability and balanced solubility–lipid solubility profiles. Most of the evaluated compounds also exhibit a low number of rotatable bonds (ranging from 0 to 4), which is typically associated with increased molecular rigidity and, consequently, better oral bioavailability. The only exception is Tetracosanoic acid, which possesses a higher number of rotatable bonds due to its long-chain fatty acid structure, potentially affecting its conformational flexibility and bioavailability.

#### 2.2.1. Pharmacokinetics of Natural Compounds Extracted from *P. nigrum* Using Deep-PK

By integrating these molecular descriptors, the analysis provided valuable insights into the pharmacokinetic suitability of the investigated compounds and facilitated the prioritisation of candidates for further pharmacodynamic evaluation and potential therapeutic development. This preliminary filtering step is a critical component of modern drug discovery pipelines, as it enables the early identification of promising lead compounds while minimising the risk of costly experimental failures in later stages of development.

Pharmacokinetic prediction plays a fundamental role in understanding the journey of a compound within the human body. Regardless of the route of administration, comprehensive ADMET (Absorption, Distribution, Metabolism, Excretion, and Toxicity) profiling is essential to evaluate the compound’s drug-like behaviour. Table 6 summarises the key pharmacokinetic parameters analysed for the selected natural compounds derived from *P. nigrum*.

From the absorption perspective, two aspects were considered: gastrointestinal absorption and transdermal absorption. While gastrointestinal absorption is routinely assessed, this study also aimed to evaluate the potential of the compounds for absorption through the skin, which is particularly relevant for topical or transdermal therapeutic applications.

About distribution, a critical factor is the ability of compounds to cross the blood–brain barrier (BBB). Compounds capable of penetrating the BBB may exert central nervous system (CNS) activity or side effects, which must be considered depending on the therapeutic goal.

Special attention was given to the potential inhibition of the organic cation transporter 2 (OCT2), which plays a crucial role in renal excretion. Compounds that are non-inhibitors of OCT2 and exhibit a relatively favourable half-life are considered more suitable for further development, as they are less likely to accumulate in tissues and cause adverse effects.

According to predictions from the Deep-PK database, all of the analysed compounds are expected to be well absorbed in the human intestine, with six of them showing high absorption probabilities (>0.9), indicating strong potential for oral bioavailability. Regarding transdermal (skin) absorption, Piperine (−2.08), Piperolactam A (−1.30), Piperolactam D (−1.85), and Cepharadione A (−1.90) demonstrated more favourable absorption scores compared to Tetracosanoic acid (−3.05), 3,4-Methylenedioxycinnamic acid (−2.74), 2-Butenedioic acid (−3.21), and Piperonylic acid (−3.28), suggesting a better potential for skin permeability among the first group.

In terms of distribution, seven out of the eight compounds are predicted to cross the blood–brain barrier (BBB) with high confidence, showing probabilities ranging from 0.779 to 1.000. The only exception is Piperolactam D, which is classified as non-penetrable, with a lower confidence score of 0.26, indicating limited central nervous system accessibility.

Cytochrome P450 enzyme interaction predictions revealed several notable findings. Piperolactam A, Piperolactam D, and Cepharadione A are predicted to act both as inhibitors and substrates of CYP1A2. Additionally, these three compounds, along with Tetracosanoic acid, are predicted to inhibit CYP2C19. Cepharadione A is the only compound identified as an inhibitor of CYP2C9 and, together with Piperine, may also act as an inhibitor of CYP2D6 and CYP3A4.

In terms of substrate classification, Piperolactam A, Cepharadione A, and 3,4-Methylenedioxycinnamic acid are predicted to be substrates of CYP2C9, while Piperine is the only compound predicted as a substrate for CYP2D6. None of the compounds were predicted to act as substrates for CYP2C19 or CYP3A4, which may reduce the risk of metabolic competition or interaction at those specific isoenzymes.

These predictions provide valuable insights into the absorption, distribution, metabolism, and potential drug–drug interaction profiles of the studied *P. nigrum* compounds, which are essential for guiding further pharmacological and experimental investigations.

When it comes to in silico toxicity (Table 7), hepatotoxicity is the main issue, as multiple compounds are predicted to cause liver injury with high probability (0.983–0.502). Piperolactam A, Piperolactam D and Cepharadione A are reported to have mutagenic potential (AMES test), although all compounds are predicted as non-carcinogenic. Tetracosanoic acid and 2-Butenedioic acid are the only two molecules predicted as eye corrosive. Tetracosanoic acid is also the only one predicted as cardiotoxic (hERG blocker).

#### 2.2.2. Pharmacodynamics of Natural Compounds Extracted from *P. nigrum*

To predict the pharmacodynamic properties of the natural compounds extracted from *P. nigrum* (Table 8), we used the SwissTargetPrediction platform (http://www.swisstargetprediction.ch/). The analysis revealed that Piperine may act as a potential ligand for monoamine oxidase B (MAO-B), suggesting possible neurological or neuroprotective activity. Piperolactam A was predicted to interact with cyclin-dependent kinase 2 (CDK2), indicating potential involvement in cell cycle regulation. Additionally, Tetracosanoic acid may interact with the fatty acid-binding protein adipocyte (FABP4), which could suggest a role in lipid metabolism or adipogenesis.

Fatty acid binding proteins are important molecules in metabolic and inflammatory pathways. Although they are highly conserved proteins, little is known about the mechanisms of action of these proteins, but they are known to influence the import, storage and export of fatty acids, as well as the metabolism of cholesterol and phospholipids [63].

Cyclin-dependent kinases (CDKs) constitute a family of regulatory proteins that play essential roles in cell cycle progression, cellular proliferation, and gene expression. In addition to these functions, CDKs act as signalling hubs, integrating endogenous and exogenous stimuli. The interaction of natural compounds with CDKs may influence their activity by modulating substrate proteins, thereby enhancing cellular processes such as proliferation and repair, ultimately contributing to accelerated wound healing [64,65].

Monoamine oxidase is an enzyme involved in the degradation of neurotransmitters. It has a wide distribution in the human brain. Monoamine oxidase inhibitors could have pharmacological effects against various neurological disorders, thus having increased therapeutic importance [66].

### 2.3. Powder Formulation

**Figure 3:**
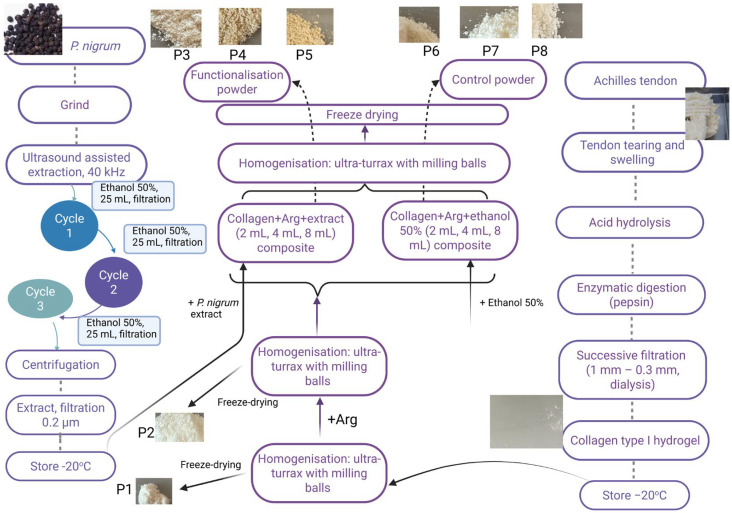
Formulation scheme and appearance of collagen–arginine powders functionalized with a hydroalcoholic extract of *P. nigrum*.

Compared to conventional dressings, powders have several benefits. These can be applied directly to the injured surface, conforming evenly to the shape and size of the wound. Dried products are less susceptible to bacterial contamination and have a longer shelf life without requiring special storage conditions [10,67]. Upon contact with wound fluids, the powders rehydrate, forming a barrier that absorbs exudate and gradually releases the bioactive compounds. Additionally, because they allow for precise dosing and can be combined with other medications, they are more versatile than conventional dressings [10]. The formulation protocol for type I collagen-based composites functionalised with *P. nigrum* extract and arginine (Arg) is presented in Figure 3. The *P. nigrum* extract was obtained by UAE and combined with type I collagen extracted from bovine Achilles tendons (according to the method described by [68] and characterised by [69]. To ensure the formation of a powder, Arg, acting as a stiffening agent, was added, which transforms the collagen gel into a solid material suitable for handling and easy topical application. Arg is a basic amino acid, positively charged at physiological pH [70]. It can form ionic and hydrogen bonds with the carboxyl groups in collagen, thus stabilising the network [71]. These interactions can increase the crosslinking capacity, making the structure more compact, which helps in the formation and stabilisation of powders after freeze-drying [72]. It can influence the hydration and rehydration of powders, which is important for topical applications where the material needs to be easily reactivated by increasing the solubility of the final powders [73]. In addition to its structural role, Arg is a substrate for the synthesis of nitric oxide (NO), a biomarker that stimulates vasodilation, angiogenesis, and epithelialization [74], ensures skin regeneration by supporting the synthesis of endogenous collagen and fibroblast proliferation [75], and has an anti-inflammatory effect through the production of NO and modulation of the immune response [76]. In this study, the effect of *P. nigrum* extract incorporated in collagen-Arg composite and cefazoline was evaluated. Arg provides cationic groups that can interact electrostatically with bioactive molecules, facilitating their encapsulation or retention within the matrix and thereby enhancing the antioxidant and antimicrobial effects of currently used antibiotics in wound infections.

#### 2.3.1. Physico-Chemical Characterisation of Powders

**Figure 4:**
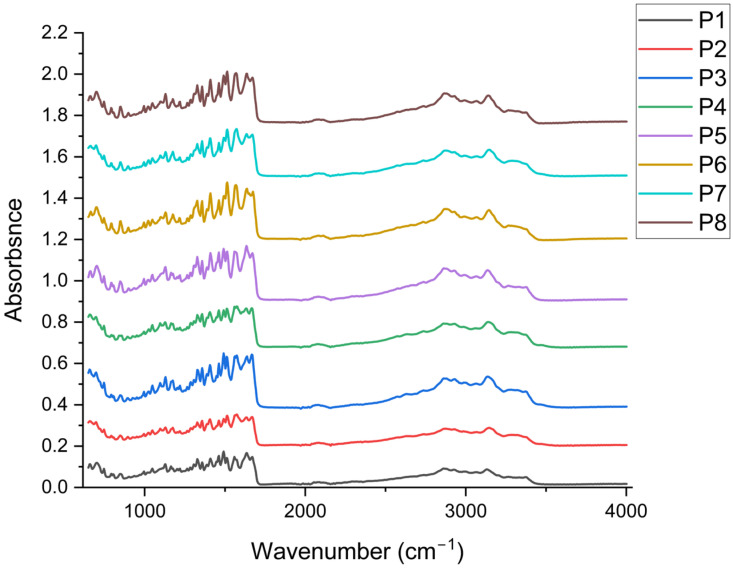
Comparative FTIR spectra of samples P1–P8 highlighting the effect of arginine, *P. nigrum* extract and ethanol on the collagen matrix (n = 3).

The collagen control (P1) provides spectral references for identifying the main changes in collagen structure at each formulation stage (Figure 4). Amide A, represented by the broad ν(O–H, N–H) band in the 3300–3500 cm^−1^ region, is attributed to hydrogen bonding in hydrated collagen [77,78]. The specific Amide I band, located at wavenumbers between 1674–1637 cm^−1^, is specific to ν(C=O), a band sensitive to the triple helix conformation [79,80]. Amide II is specific to the wavenumber 1562 cm^−1^ [81]. The presence of Amide III in the range of 1331–1268 cm^−1^ (P1 has 1331, 1309/1287–1268 cm^−1^) indicates the preservation of the collagen triple helix [82]. In the aliphatic region, the stretching bands ν(C–H) at 2988, 2925, and 2862 cm^−1^ are prominent, while in the “fingerprint” region, absorption maxima appear at 1208, 1167, 1134, 1100, 1052, and ~1000 cm^−1^. These bands can be attributed as follows: (i) 2988–2862 cm^−1^ (ν_as/s CH_3_/CH_2_) of the aliphatic chains of collagen and organic components in the matrix [83], (ii) 1208–1167 cm^−1^ (ν(C–N), ν(C–O), δ(N–H)) from mixed Amide III/polyol contributions [77], (iii) 1134–1100 cm^−1^ (ν(C–O), ν(C–O–C)) specific to ether/alcohol bonds (proteins, solvent traces/polysaccharides) [84,85], and (iv) 1052–1000 cm^−1^ (ν(C–O) secondary, δ(C–H)) from typical vibrations for glycosylated phenol fragments and the protein backbone [86].

By including arginine (P2), which stabilised the protein matrix during freeze-drying, leading to the formation of solid, compact structures that subsequently allowed for the production of powders and is present in all P3–P8 (Figure 4). In P2, slight shifts toward lower wavenumbers are observed in the N–H/O–H stretching bands (e.g., 3372, 3283/3268 cm^−1^) and the Amide II shift from 1562 (P1) to 1577 cm^−1^, indicating the strengthening of the H-bonding network and the interaction of guanidinium (arginine) with the peptide chain [87,88,89]. In the Amide I region, discrete variations (1674–1670–1678 cm^−1^) without the loss of the Amide III band (1331/1268 cm^−1^) ensure the preservation of the triple-helix structure [90].

Adding *P. nigrum* extract (P3–P5; 2–8 mL) ensured the appearance/enhancement of aromatic signatures. In P3–P5, the typical bands of the aromatic skeleton are enhanced, such as: 1514 cm^−1^ (P3, P4, P5) compared to 1518 cm^−1^ in the samples without *P. nigrum* extract (P2, P6–P8), attributed to the vibrations of the benzene ring (C=C) [91], as well as enhancements for the specific bands at wavenumbers 1000–1030 cm^−1^ (P3–P5: 1030/1026 cm^−1^) associated with the ν(C–O)/δ(C–H) vibrations of phenols [92]. Out-of-plane C–H bending vibrations of aromatic rings (955, 855, 810 cm^−1^) become more pronounced in P3–P5, suggesting the contribution of specific aromatic compounds [93,94] in the extract (phenolic acids, flavonoids). Concurrently, Amide II remains around 1577 cm^−1^ (P3–P5), and Amide I maintains its maximum absorption at ~1670–1674/1637 cm^−1^ without any collagen denaturation being observed [95]. With increasing extract dose (P3→P5), the aromatic bands and those in the 1026–1030 cm^−1^ region tend to be more prominent, in accordance with the higher concentration of phenolic compounds. It is noted that the bands at 1309 cm^−1^, 1391 cm^−1^, and 1562 cm^−1^ were highlighted exclusively in the samples containing *P. nigrum* extract, suggesting the contribution of phenolic/aromatic compounds to these vibrations [96,97,98]. Their appearance indicates both additional interactions between the phenolic groups and the collagen matrix, as well as possible overlaps with the characteristic Amide II and Amide III bands.

Possible changes caused by the solvent (50% EtOH: P6–P8, 2–8 mL) used for each sample with extract were determined. Samples P6–P8 (without *P. nigrum* extract) exhibit slightly different C–H patterns (e.g., 2992/2929 cm^−1^ in P6–P8 vs. 2988/2925–2933 cm^−1^ in other samples) and the appearance of 1104 cm^−1^ (P6, P8) attributed to C–O of alcohols/polysaccharides [99]; however, the typical aromatic traces (1514, 1030, 955, 855, 810 cm^−1^) observed in P3–P5 are missing. The bands for Amides I/II/III remain practically in the same positions as in P2, indicating that 50% EtOH does not visibly denature the collagen under the experimental conditions.

Collagen’s structural signatures (stable Amide I bands ~1674/1637, Amide II ~1562–1577, and Amide III ~1331/1268 cm^−1^) are preserved in all samples, according to FTIR spectra. The formulations containing arginine showed slight shifts in the ν (N–H/O–H) and Amide II bands, indicating a consolidated hydrogen bond network without the loss of the triple helix. The *P. nigrum* extract (P3-P5) formulations show distinct phenolic bands (≈1514 and 1030–1026 cm^−1^) and enhanced out-of-plane band area (907–795 cm^−1^), indicating phenolic compound encapsulation and non-covalent interactions with the protein matrix. It is confirmed that the changes seen in P3–P5 are due to the extract and not the solvent because the series with 50% EtOH (P6–P8) only exhibits slight adaptations of the C–H/C–O bands (2992/2929 cm^−1^ and 1104 cm^−1^), with no rise in the aromatic signatures. With the retention of the collagen structure and the addition of phenolic compounds proportionate to the extract dosage, the FTIR analysis generally confirms the compatibility between collagen–arginine and the *P. nigrum* extract.

TGA-DSC analysis was undertaken to test if the chemical interactions predicted by FTIR translate into stability changes, which confirms a dose-dependent thermal stabilisation of the network upon the incorporation of *P. nigrum* extract. The mass losses and temperatures of the thermal effects were evaluated for samples P1–P8 using TG–DSC analysis.

**Figure 5:**
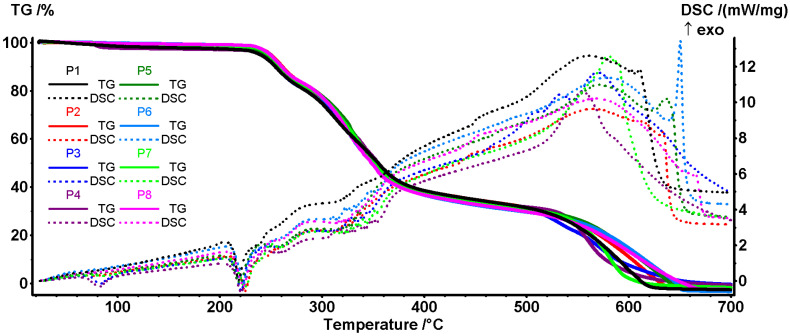
Integrated thermal analysis (TGA–DSC) of collagen–arginine composites with/without *P. nigrum* extract (P1–P8): TG/DSC curves and derived parameters (mass loss and endo/exo peak temperatures). The exothermic effects are pointing upwards.

In the first temperature interval (RT–210 °C), samples exhibited a small mass loss between 1.05–3.11% (Figure 5 and Table 9), assigned to residual solvent desorption and collagen denaturation, with samples P3 and P4 having the highest mass loss. At this temperature, the most volatile components from extract are also eliminated [68,100]. On the DSC curves, this mass loss is accompanied by a weak endothermic effect at 68–83 °C, sustaining the assignments made [101]. The higher values (82–83 °C), observed for samples P3, P4, and P7, are related to the higher mass loss recorded in this temperature interval. The samples’ degradation continues in the second temperature interval, 210–280 °C, when a mass loss of ~15–16% is recorded. On the DSC curve, the process is accompanied by a characteristic endothermic effect at ~220 °C, related to the transition from the collagen triple-helix structure to random twisted chains [101]. Polymeric chains start to degrade after this change, as indicated by the increasing mass loss rate. The backbones are fragmented and partial oxidation processes occur; the endothermic and exothermic effects overlap after 230 °C. Among possible reactions are collagen pyrolysis, including the cleavage of peptide bonds and deamination/decarboxylation of side chains [102]. After 280 °C the oxidation reactions become predominant, as indicated by the DSC effects, as the main pyrolysis of collagen/organic matrix occurs, with the mass loss ranging between 48 and 52%. Sample P3, the one with the lowest extract concentration, shows values similar to the control collagen (P1). By increasing the extract concentration and mass loss, the values increase, but to a lesser extent compared to the ethanol-collagen controls (P6–P8), indicating a higher carbon residue yield. In the fourth stage (470–700 °C), the complete burn of the residual carbonaceous mass takes place, with mass losses observed between 33.80 and 34.67, and the broad and strong exothermic peak in the range of ~555–580 °C [103].

All samples are relatively stable up to 210 °C (minimal losses), so common processes involving temperatures below 150 °C (such as drying, handling, sterilisation with EtOH/UV) do not significantly affect the matrix. The *P. nigrum* extract did not change the degradation mechanism, but the minor differences in mass loss % and position of the thermal effects may suggest that it strengthens the helix (possible H bonds/phenolic cross-linked collagen) [104,105].

**Figure 6:**
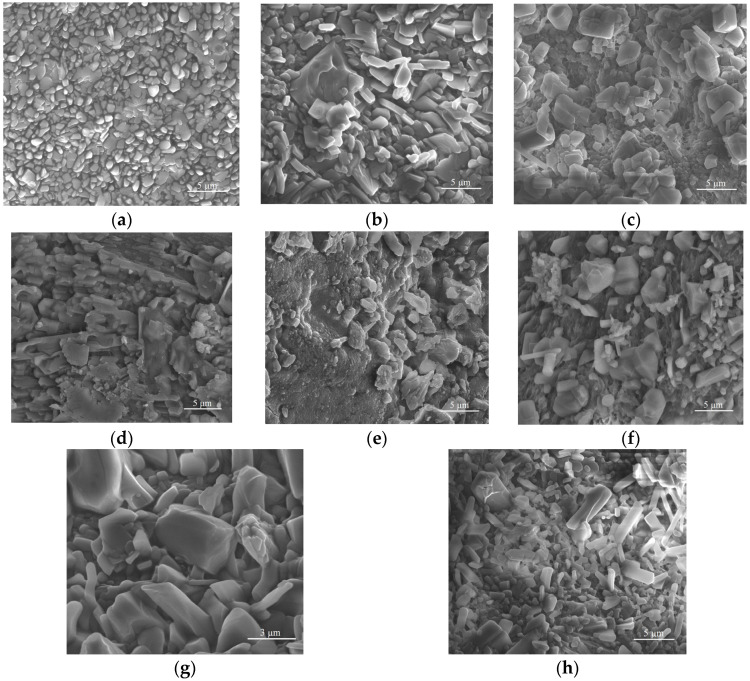
SEM images of collagen–arginine composites with/without *P. nigrum* extract (P1–P8): evolution of microstructure depending on composition: (**a**) Collagen (P1), (**b**) Collagen + Arginine (P2), (**c**) Collagen + Arginine + 2 mL *P. nigrum* extract (P3), (**d**) Collagen + Arginine + 4 mL *P. nigrum* extract (P4), (**e**) Collagen + Arginine + 8 mL *P. nigrum* extract (P5), (**f**) Collagen + Arginine + 2 mL EtOH (P6), (**g**) Collagen + Arginine + 4 mL EtOH (P7), (**h**) Collagen + Arginine + 8 mL EtOH (P8).

The microstructure of the crushed and subsequently freeze-dried collagen particles was also evaluated by SEM (Figure 6). The obtained micrographs clearly showed that the collagen particles assemble into irregularly shaped spheroids similar to the forms obtained by Dang et al. [106]. The particles of native collagen that have been triturated, freeze-dried, and subsequently ground (Figure 6a) exhibit relatively uniform, granular-compact surfaces with small particles adhering to each other. Fine texture, without large separate domains, providing a homogeneous matrix. Figure 6b shows the micrograph for the collagen powder with arginine (P2), where larger plates/facets and more pronounced edges, resembling irregularly arranged crystals, can be observed. Roughness increases compared to P1; likely areas rich in crystallised arginine within the collagen mass. In the case of the samples functionalised with *P. nigrum* extract (2 mL of extract in Figure 6c; 4 mL of extract in Figure 6d; 8 mL in Figure 6e), an amorphous-granular coating was visible over the base particles and small dispersed agglomerates; the distribution appears quite uniform. No obvious microcracks are appearing, which may suggest good integration at a low dose. At the average extract concentration, the plates become more “rounded” or fused, the surface appears denser, and the contiguous domains are covered by the organic phase of the extract. It indicates increased compatibility and the beginning of phase coalescence. The highest extract concentration led to increased roughness and the formation of large organic phase agglomerates; in some areas, a sticky appearance was observed, indicating matrix overload with extract. This neo-homogeneity can explain why, in the evaluation of antimicrobial activity by the diffusion method, high doses do not yield maximum synergy (weaker diffusion through aggregates).

For the controls with similar volumes of 50% ethanol (Figure 6f–h), the morphology appeared similar to P2, with well-defined particles. No organic film/nobbling was observed as in P3–P5, which confirms that the solvent alone did not significantly alter the morphology compared to P2.

Therefore, P1 exhibits a granular-compact surface, relatively homogeneous (Figure 6a). The addition of arginine (P2) led to the formation of more defined particles or plate-like structures, accompanied by a slight increase in surface roughness (Figure 6b). Incorporation of *P. nigrum* extract (P3 and P4) produced a uniformly distributed irregular–granular morphology throughout the matrix, indicative of good compatibility at low to medium concentrations (Figure 6c–e). At the highest concentration (P5), the organic phase exhibited clumping and/or coalescence, suggesting overloading and local inhomogeneity. In contrast, the ethanol controls (P6–P8) retained a faceted-granular morphology similar to P2, without the amorphous film characteristic of the extract-containing samples, confirming that the observed morphological changes were attributable to the *P. nigrum* compounds rather than the solvent (Figure 6f–h).

**Figure 7:**
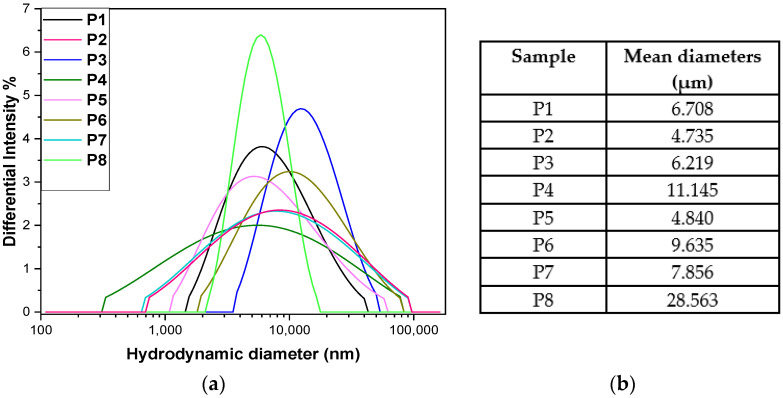
DLS hydrodynamic diameter profiles (**a**) and corresponding mean particle sizes for samples P1–P8 (**b**) (n = 3).

DLS analysis was used to evaluate the size and degree of aggregation of the suspended particles, highlighting the stability and interactions between the molecules (Figure 7a). The size of native collagen particles was relatively large [105,107] but stable (P1). Collagen tends to form aggregates in solution due to intermolecular interactions and the lack of a strong stabiliser. Adding Arg reduces the size of the aggregates, suggesting that Arg partially stabilises collagen through electrostatic interactions (Arg is a positively charged molecule with the potential to reduce clumping).

The size increases compared to P2 by including a 2 mL volume of *P. nigrum* extract (P3), thus introducing polyphenolic compounds or alkaloids that can favour hydrophobic interactions with collagen, leading to the formation of larger aggregates. In test P4, the size increase was more pronounced, likely at a higher concentration, pepper promotes aggregation by creating hydrophobic bridges and multiple interactions with the protein. By including a larger volume of extract (P5), the size decreases sharply, almost to the value of P2 (Figure 7b). This can be explained by the fact that at high concentrations, the compounds in pepper act as natural surfactants and prevent aggregation (a stabilising effect after a critical concentration) or induce partial hydrolysis of collagen.

Ethanol promotes protein dehydration and aggregation (P6–P8), which explains the higher values compared to P2. The P7 sample showed a slight decrease compared to P6. At moderate concentrations, ethanol can destabilise hydrophobic interactions and reduce aggregation, while at a higher volume of ethanol, a massive increase was observed, likely due to the ethanol precipitating the collagen, leading to very large, difficult-to-disperse aggregates. This is a classic effect of alcohol on proteins, revealing the highest hydrodynamic diameter [108].

#### 2.3.2. Influence of Phenolic and Flavonoid Content on the Antioxidant Capacity of Collagen Composites with *P. nigrum* Extract

**Figure 8:**
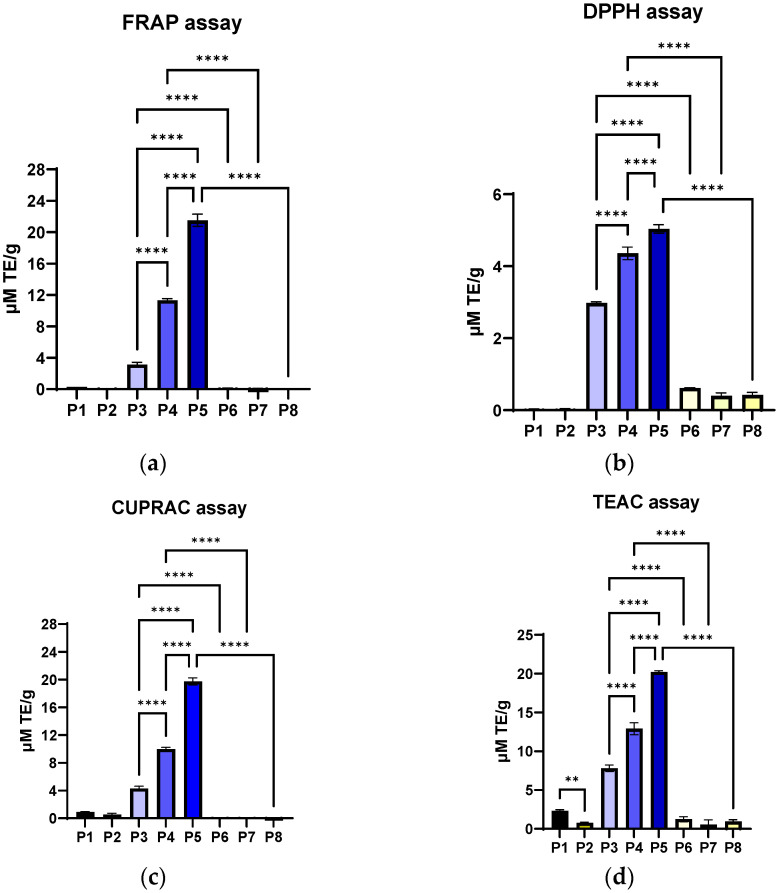
Antioxidant activity of Col–Arg composites functionalized with *P. nigrum* extract analysed by the following methods: (**a**) DPPH, (**b**) FRAP, (**c**) CUPRAC, and (**d**) TEAC assays, expressed as µM TE/g. Groups: P1 collagen; P2 collagen–arginine; P3/P4/P5 collagen–arginine-*P. nigrum* extract (2/4/8 mL); P6/P7/P8 collagen–arginine-EtOH (2/4/8 mL). Values are expressed as mean ± SD, n = 3; statistical significance analysed by one-way ANOVA (Tukey’s test) is indicated on the graphs (** *p* < 0.01; **** *p* < 0.0001).

The inclusion of *P. nigrum* extract caused a clear, dose-dependent increase in the DPPH radical scavenging effect. The increase was almost linear with increasing extract concentration, indicating that the bioactive compounds in *P. nigrum* were responsible for neutralising the radicals (Figure 8a). Collagen and arginine, whether alone or combined, do not significantly contribute to the neutralisation of DPPH radicals. This suggests that the protein base has a structural role but not a direct antioxidant one. Adding ethanol in volumes equivalent to the *P. nigrum* extract did not alter the material’s functional properties or affect its structural integrity. This confirmed that the effect observed in P3–P5 was strictly attributed to the bioactive compounds from *P. nigrum*. The fact that all differences between extract and controls were significant (*p* < 0.0001) indicates the robustness of the effect and supports a solid dose–response relationship.

In the FRAP test (Figure 8b), the maximum values (P5) were almost 2–4 times higher than those obtained by the DPPH method. This suggests that *P. nigrum* extract had a stronger metal ion reducing capacity than direct free radical scavenging ability. In other words, in this type of matrix (collagen + arginine), the antioxidant effect of the *P. nigrum* extract was more evident through the reducing pathway. Materials P1 and P2 confirm that the protein base does not offer direct antioxidant benefits, and P6–P8 (ethanol-equivalent volume controls) exclude the influence of the solvent, strengthening the idea that the effect comes solely from *P. nigrum*.

The CUPRAC profile (Figure 8c) was very similar to FRAP. The values obtained by the CUPRAC method (P5: 19.73 ± 0.52 µM TE/g) are almost identical to those from FRAP (P5: 21.51 ± 0.78 µM TE/g), which indicates that the *P. nigrum* extract had a consistent capacity to reduce transition metals (Fe^3+^ and Cu^2+^). This indicates an antioxidant mechanism based primarily on electron donation and the reduction of metal ions, rather than simply radical scavenging (as was the case with the DPPH assay).

TEAC is based on the ABTS radical scavenger (Figure 8d), which measures the total antioxidant capacity (both electron donation and radical scavenging) [109]. The values obtained are comparable to FRAP and CUPRAC but significantly higher than DPPH. The fact that the TEAC values (P5: 20.21 ± 0.18 µM TE/g) align with CUPRAC and FRAP assays and are much higher than those obtained by the DPPH method suggests that, overall, the antioxidant effect of the *P. nigrum* extract was predominantly based on electron donation, not direct free radical scavenging. Collagen alone (P1) has a very small but statistically detectable effect (*p* < 0.01), possibly due to some amino acids with weak antioxidant potential [110] being inactivated by the inclusion of arginine. However, its contribution was insignificant compared to the massive effect of the pepper extract.

For the ensemble, Col-Arg has minimal contributions, and ethanol does not influence the results. CUPRAC and TEAC assays confirm the FRAP results: the *P. nigrum* extract was responsible for a strong and dose-dependent metal ion reducing capacity. This showed that the phenols and alkaloids in *P. nigrum* serve as electron donors and reducing agents [111,112], which explains the high results in these assays. The similarity of the three approaches implies that the antioxidant effect was relevant and precludes the role of other variables (collagen, arginine, solvent).

**Figure 9:**
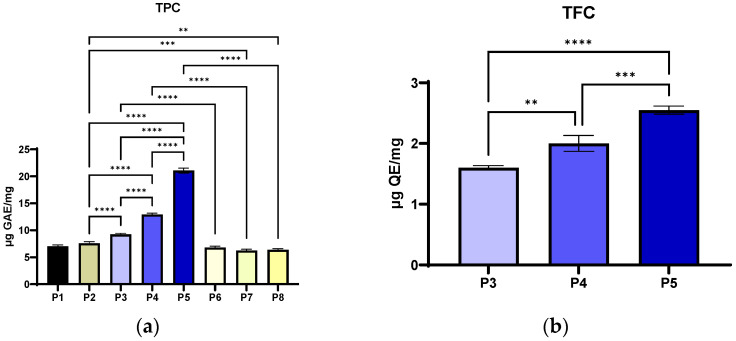
Phenolic and flavonoid content of Col–Arg composites functionalized with *P. nigrum* extract: (**a**) TPC—total phenolic content determined by Folin–Ciocalteu (µg GAE/mg) for P1 collagen; P2 collagen-arginine; P3/P4/P5 collagen-arginine-*P. nigrum* extract (2/4/8 mL); P6/P7/P8 collagen-arginine-EtOH (2/4/8 mL); (**b**) TFC—total flavonoid content (µg QE/mg) for samples with *P. nigrum* extract (P3–P5). Values are expressed as mean ± SD; statistical significance was analyzed using the one-way ANOVA method (Tukey's), indicated in the graphs as ** *p* < 0.01, *** *p* < 0.001 and **** *p* < 0.0001.

To evaluate the total polyphenol content and, implicitly, the exact content, the collagen powders were solubilised in PBS (pH 7.4). The materials are completely dissolved in PBS, thus excluding the retention of the active substance in the undissolved material, meaning that the differences between the samples reflect the composition, not the dissolution yield. From Figure 9a, it can be observed that the TPC increases with the concentration of extract included in the collagen material (P3 < P4 < P5). The differences between the collagen materials with *P. nigrum* extract and the similar ethanol controls (P3 vs. P6, P4 vs. P7, P5 vs. P8) were significant (*p* < 0.0001). The differences were also found to be significant between the extract-functionalised materials (P3 vs. P4 vs. P5, *p* < 0.0001). Materials P1 (collagen) and P2 (collagen with Arg) had low to moderate TPC (7.03 ± 0.25 µg GAE/mg, 7.59 ± 0.32 µg GAE/mg), but significantly lower compared to P3–P5. According to Figure 9a, it can be observed that the Folin–Ciocalteu reaction was not 100% selective for phenols, as collagen controls without extract contain reducing amino acids [113,114] that contribute to the signal (P1, P2 and P6–P8).

The TPC level increases with the dose extract and correlates well with all the data obtained for DPPH, FRAP, CUPRAC and TEAC assays. This supports the conclusion that the main mechanism is an electron donor, typical for phenolic compounds in *P. nigrum*, which predominates. The TPC determination revealed a significant, dose-dependent increase in the phenolic compound content in the samples with *P. nigrum* (P3–P5), with a maximum in P5. The low values of the control samples (P1 and P2) and the ethanol-without-pepper extract samples (P6–P8) suggest that the solvent and protein matrix have a negligible effect on the Folin–Ciocalteu signal.

The determination of TFC revealed a significant, dose-dependent increase in the samples with *P. nigrum* (P3–P5), with a maximum in P5 (Figure 9b). The chromatographic profile shows the presence of the flavonoids rutin, isorhamnetin, and dihydrochalcones (phloretin/phlorizin), whose structure with a catechol and 3-OH/4-oxo group confers a high electron-donating capacity and metal ion chelating ability [115,116]. According to the correlation with the FRAP/CUPRAC/TEAC results, the extract’s overall antioxidant mechanism was mostly redox (donating electrons), and the solvent or matrix’s contribution was minimal, with levels below the detection limit of the assay. The extract contains flavonoids such as rutin, isorhamnetin, phloretin, phlorizin, vitexin, and epicatechin/catechin. Rutin and isorhamnetin (quercetin core) have a catechol B-ring and a 3-OH/4-oxo system [117], making them electron donors and metal chelators, which explains the high FRAP/CUPRAC values and why TEAC aligns with them. Dihydrochalcones (phloretin/phlorizin) contribute more to ABTS (TEAC) and less to DPPH, according to Platzer et al. [118]. Glycosylation (rutin, vitexin, phlorizin) can reduce reactivity by DPPH (steric/lipophilic), but it affects FRAP/CUPRAC much less [119].

Numerically, TFC is a fraction of TPC (12.1% of TPC for P5, 15.50% of TPC for P4, 17.32% of TPC for P3), so non-flavonoid phenols (and other Folin-active reductants) are the majority; however, rutin/isorhamnetin-type flavonoids have a disproportionate impact on reducing tests. The *P. nigrum* extract brings a real increase in TFC, proportional to the dosage, and the structure of the major flavonoids in the extract (rutin, isorhamnetin and dihydrochalcone) perfectly explains why the antioxidant activity measured by the FRAP, CUPRAC, and TEAC methods was high.

#### 2.3.3. Study of Antimicrobial Synergy Between *P. nigrum* Extract and Antibiotics

Cefazolin, a first-generation cephalosporin, is effective against Gram-positive bacteria, including *S. aureus* and β-haemolytic streptococci, which are common causes of skin and soft tissue infections [120]. Bacterial colonisation and biofilm formation (particularly by *S. aureus* and *S. epidermidis*) are important causes of wound closure delays. When used against these bacteria, cefazolin effectively lowers the microbial burden and stops the illness from spreading [53]. Cefazolin has a number of advantages over other antibiotics, including: (i) a good safety profile and clinical tolerability; (ii) effective tissue penetration, including into the skin and subcutaneous tissue, making it useful in treating wound infections both locally and systemically; and (iii) parenteral administration, which ensures quick and efficient concentrations at the infection site [24,26,27,28]. The use of cefazolin in the context of chronic wounds can be justified both as an initial empirical treatment for Gram-positive bacterial infections and as an adjuvant agent in combination with other strategies (e.g., natural agents with antibiofilm effects, local therapies). Cefazolin is very effective against Gram-positive bacteria, but has a limited spectrum against Gram-negative bacteria [29].

The functionalised collagen material with *P. nigrum* extract (MF) alone has an inhibition diameter of 0 mm, with a single exception: *S. epidermidis* ATCC 12228 (P3—the material with the lowest concentration of extract) 15.00 ± 1.41 mm; practically, the extract has intrinsic activity against this coagulase-negative *Staphylococcus* (Table 10). In combination with cefazolin (KZ), the effect depends on the species and the sample (P3–P8). The effect was considered synergistic only if the difference between the diameter of the KZ and that of the combination with the functionalised material was greater than or equal to 2 mm.

For Gram-positive bacteria, a consistent synergistic effect was observed in most samples (Δ = 2–4 mm for P3–P6, P8, P7) against the *S. aureus* ATCC 25923 strain, while against *MRSA* the relationship was indifferent (Δ = 0–1.5 mm), and the non-functionalised control (P7) even showed a slight antagonism (−0.5 mm). For the *S. aureus* strain (clinical isolate), no synergistic effects were observed (Δ ≤ 2 mm), while the control material (P6) proved to be strongly antagonistic (−5.5 mm). In the case of the *E. faecalis* strain, the material led to the inactivation of the antibiotic (KZ), with strong antagonism being observed at P3–P7. Enterococci have intrinsic resistance to cephalosporins [121], and the matrix/extract does not seem to help (possible interaction with the antibiotic or with diffusion in the agar). A synergistic effect was observed for P3–P5 (Δ 3–4 mm) against the *S. epidermidis* strain, while for the control materials, the effects are borderline.

In the case of Gram-negative bacteria, strains for which KZ exhibits resistance [122,123] showed synergy in the case of *E. coli* strains, but the magnitude of the synergy increases as the extract concentration decreases. This behaviour reflects diffusion limitations and/or physicochemical interactions of collagen powders in agar at high loadings, as well as adaptive responses of the outer membrane (reduction of porins, increased efflux, possible induction of β-lactamase) [124,125]. The best synergistic effect for both *E. coli* strains studied was for sample P4. In the case of *P. aeruginosa* and *K. pneumoniae* strains, only the strains isolated from infected wounds showed synergism in the presence of KZ, with P4 having the best results for the *P. aeruginosa* strain (Δ = 3.5 mm), while for *K. pneumoniae* it was P3 (Δ = 3 mm). In the case of *P. mirabilis* strains, the same effect was observed: materials with the lowest concentration of extracts showed a better synergistic effect with KZ, with variant P3 (Δ = 3.5 mm for *P. mirabilis* ATCC 27853, Δ = 2.5 mm for *P. mirabilis* 11P) having the best effect. For validation, for the variants that generated synergy through the qualitative method, a checkerboard (FICI) was performed to confirm and validate through quantitative methods.

Chronic wounds (diabetic ulcers, pressure sores, venous ulcers) are frequently colonised or infected with Gram-positive bacteria, particularly *S. aureus* (including MRSA), *S. epidermidis*, and *E. faecalis*. These infections delay healing and complicate treatment, but prolonged use of antibiotics (including cefazolin) can lead to resistance or reduced effectiveness. Consequently, techniques that integrate antibiotics with natural or bioactive extracts are undergoing rigorous investigation.

According to Table 11, powders P3 (2 mL) and P4 (4 mL) showed an additive effect with cefazolin against *S. aureus* ATCC 25923, *S. aureus* sc pl, and MRSA. At higher concentrations (P5, 8 mL), the effect was reduced to indifference, suggesting that optimal doses were small to medium. The effect of the functional powders against *S. epidermidis* and *E. faecalis* was rather indifferent or antagonistic, which indicates that the benefit of the *P. nigrum* extract was specific to staphylococci. Similar controls containing ethanol showed that low-to-medium doses (P6, P7) had an additive effect only against *MRSA*; otherwise, the effect can be attributed to the *P. nigrum* extract and not the solvent used. At high doses (P8), antagonism occurs, especially with *E. faecalis.* So, the ethanol control (P6–P8) did not really add to the antibacterial activity, and in high doses, it can reduce the antibiotic’s activity. Low doses of cefazolin with *P. nigrum* can effectively treat *S. aureus* infections in chronic wounds. However, it is not recommended for *E. faecalis*-dominated infections due to antagonism. The additive action of *P. nigrum* at low doses and antagonism against *E. faecalis* were confirmed by both tests for *S. aureus* ATCC 25923, *S. aureus* sc pl, and *E. faecalis*. The quantitative method showed additivity, whereas diffusion only showed indifference in the case of *MRSA* or synergism in the case of *S. epidermidis*, as indicated by the differences in the findings obtained for the two bacteria. These differences can be explained by the constraints of each approach, as the quantitative method emphasises molecular interactions in a liquid medium, whilst the diffusion technique depends on the solubility and movement of chemical substances in agar.

Gram-negative bacteria, including *E. coli*, *P. aeruginosa*, *K. pneumoniae*, and *P. mirabilis*, were commonly found in chronic wounds [126]. These bacteria are challenging to treat because they can form biofilms and are resistant to antibiotics. Cefazolin’s ability to combat Gram-negative bacteria is restricted [122]; however, its effectiveness is increased when combined with natural extracts. All of the compounds produced synergism with KZ against the *E. coli* ATCC 25922 strain, indicating that the combination has a good likelihood of working in infections involving sensitive strains. For the clinical strain (*E. coli* C10E), the effect was additive, which means that the benefit was limited in resistant clinical strains. The MIC value for the uncombined materials with KZ was lowest for samples P3–P5, settling at 625 µg/mL. The *P. aeruginosa* and *K. pneumoniae* reference strains were found to be resistant to the tested doses, showing MICs that exceeded the experimental window, according to the qualitative screening. The lowest MIC values were recorded for samples P3 and P4, which contain low and medium concentrations of *P. nigrum* extract, corresponding to synergistic or additive effects (FICI ≤ 1). At higher concentrations of the extract (P5) or in the ethanol control samples (P6–P8), the interactions shifted toward indifference or antagonism (FICI > 1), confirming that the antibacterial activity of the composite was concentration-dependent and optimised at low doses of the extract.

According to good practices for combination tests, the calculation of the FICI was invalid when the MIC of any of the agents exceeded the highest concentration tested. For the *P. aeruginosa* strain isolated from the clinic, the synergistic or additive effects were likely due to the solvent treatment and not necessarily to the *P. nigrum* extract; however, the FICI values for P3 (0.541) are lower than those for the ethanol counterpart (P6: 0.791). In the case of the *K. pneumoniae* strain, the effect was consistently additive, which could help reduce antibiotic doses, but the *P. nigrum* extract did not influence the outcome in any way. Regarding *P. mirabilis* strains, the solvent control produced an indifferent effect, but the reference strain and the clinical isolate both showed an additive effect with KZ. Out of all the combinations, P3 (2 mL of *P. nigrum*) appeared to be the most promising solution, even against Gram-negative bacteria, demonstrating a lasting additive effect and synergism with *E. coli* ATCC 25922. Variants P5 and P8 (high doses of *P. nigrum* or ethanol) led to a synergistic effect against the *P. aeruginosa* strain, which has clinical relevance.

Against *E. coli* ATCC 25922, *P. aeruginosa* 1014, *K. pneumoniae* B1K, and *P. mirabilis*, the combinations showed additive or synergistic effects, according to the results of both quantitative and qualitative approaches, which were mostly consistent. For the *E. coli* C10E strain, there was a minor disparity between the diffusion test’s suggestion of indifference and the FICI value’s indication of additivity. This discrepancy can result from the diffusion method’s low sensitivity. In the context of chronic polymicrobial wounds, *P. nigrum* extracts at low to medium doses appear to be the most valuable for improving the effectiveness of cefazolin against Gram-negative bacteria.

Evaluating the antibiofilm effect is essential because biofilm protects bacteria from the action of antibiotics and the immune response, prolongs inflammation, and delays healing [127]. Testing guides the choice of effective combinations and dosages, reducing recurrences and the selection of resistance. Analysis normalised to KZ content showed that some combinations significantly reduce the amount of cefazolin needed to eradicate the biofilm (*S. aureus* ATCC 25923, KZ eq 0.18 vs. 0.31, *E. coli* ATCC 25922, 0.71–5.69 vs. 25 µg/mL, *Proteus* spp., 7.11–28.4 vs. 31.25–125 µg/mL). In the case of Gram-positive bacteria, particularly *S. epidermidis* and *S. aureus* sc pl, the combinations are not advantageous, while for *MRSA* cell adherence, P3 halved the KZ dose (Table 12).

In the case of Gram-negative bacteria (Table 12), P3 powder (*P. nigrum* small dose) inhibited adherence to lower KZ values for *E. coli*, *P. mirabilis*, and *P. aeruginosa*, while P4 (*P. nigrum* medium dose) reduced adherence for both *E. coli* and *P. mirabilis* strains compared to the MBEC value given by KZ. Samples P6–P8 generally have a disadvantage against problematic Gram-negative biofilms (*P. aeruginosa*, *K. pneumoniae*, and *P. mirabilis*).

Therefore, the combinations significantly reduced the dose of KZ required to eradicate the biofilm in Gram-negative isolates (*E. coli* and *P. mirabilis*), by 5–35 times, while for most Gram-positive strains, the equivalent dose increased, especially for solvent controls (P6-P8). The most promising global trial was P3, which showed the greatest reductions in KZ eq (*MRSA* 2x, *E. coli* 8–35x, *Proteus* 2–8x, benefits were also observed for *Klebsiella*/*Pseudomonas*).

### 2.4. Biocompatibility

**Figure 10:**
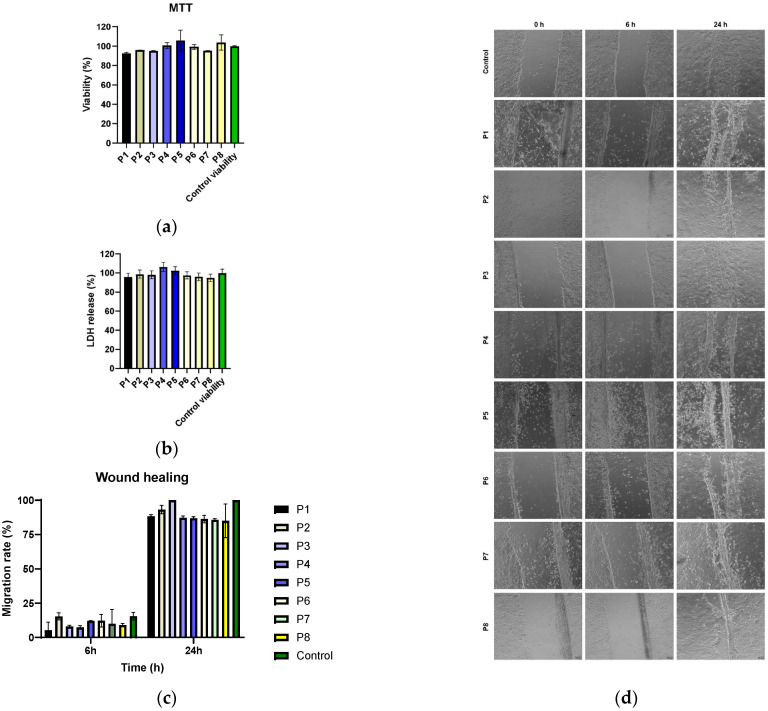
Evaluation of the biocompatibility of collagen materials on HaCaT cells using MTT (**a**), LDH release (**b**) assays and cell migration (**c**,**d**). All experiments were performed in triplicate, and the results were expressed as mean ± standard deviation. Statistical analysis was performed using the two-way ANOVA method (Sidak’s multiple comparisons test, *p* > 0.05).

The tested collagen materials (P1–P8) are biocompatible with the HaCaT cell line, showing no significant cytotoxic effect (*p* > 0.05) using the MTT method (Figure 10a). This result is important for the targeted application because it shows that *P. nigrum* extract can be used topically without negatively affecting human epithelial cells involved in the regeneration process. By evaluating the extracellular lactate dehydrogenase (LDH) content, it was found that all samples (P1–P8) had LDH release values close to the control, around 90–100% (Figure 10b). No significant increases are observed; therefore, no relevant cytotoxicity appears, and the cell membranes are intact.

The correlation between MTT and LDH indicates that the tested extracts maintain both metabolic viability and cellular membrane integrity, confirming the safety profile for application on keratinocytes. This is essential for their potential use in topical treatments for chronic wounds, where substances are needed that inhibit bacteria without harming host cells. Biocompatibility confirms that the materials have a favourable safety profile, which supports their potential as an antimicrobial/antibiofilm adjuvant in topical therapies.

The effect of the materials on the capacity of the cells to migrate and seal a wound in vitro was examined in order to assess the potential to enhance tissue regeneration. At 6 and 24 h after scratching, the uncovered area was quantified using image analysis (Figure 10d), and cell migration was assessed using the wound healing test (Figure 10c). The findings showed that all conditions had lowered migration rates at 6 h, indicating that the wound closure process was still in its early stages, since there were no appreciable variations between the samples and the control. The control group completely closed the incision within 24 h, and all groups saw a significant increase in migration rate. Samples P1–P7 showed values comparable to the control, suggesting that they do not negatively affect the healing dynamics. In contrast, sample P8 showed a decrease in the migration percentage, indicating a potential inhibitory effect on the cell regeneration process. Sample P3 (low *P. nigrum* extract concentration) had values close to the control, but they were not significantly different from the other samples (*p* > 0.05).

### 2.5. Haemocompatibility

**Figure 11:**
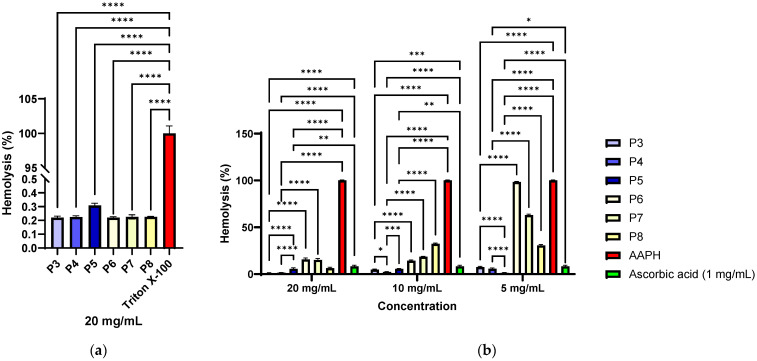
Antihaemolytic activity of materials functionalized with different concentrations of *P. nigrum* seed extract (P3–P5) compared to collagen controls without extract (P6–P8): (**a**) Haemolysis (%) induced by the extracts of samples P3–P8 and (**b**) Antihaemolytic activity of the materials against AAPH-induced oxidative haemolysis of ram erythrocytes (* *p* < 0.05, ** *p* < 0.01, *** *p* < 0.001, **** *p* < 0.0001).

Haemolysis determinations showed that all tested samples (P3–P8) had values below 1% at a concentration of 20 mg/mL, classifying them as non-haemolytic materials according to ISO 10993-4 [128] and ASTM F756 [129] standards. These results indicate that the samples do not alter the integrity of red blood cell membranes and demonstrate excellent haemocompatibility. The statistically highly significant differences observed compared to the positive control (Triton X-100, ~100% haemolysis, *p* < 0.0001) confirm that the observed effect was not non-specific but reflects the absence of an intrinsic haemolytic potential of the collagen material functionalised with *P. nigrum* extract (P3–P5, Figure 11a). Thus, the data supports the use of these materials in biomedical or dermato-cosmetic applications, where maintaining compatibility with the blood medium is an essential safety criterion.

The results demonstrate that the tested materials possess significant antihaemolytic activity against AAPH-induced oxidative stress (Figure 11b), results that correlate with those obtained through chemical methods (DPPH, FRAP, and CUPRAC). The low levels of haemolysis in the presence of the samples (below 10% at a concentration of 20 mg/mL) compared to the positive control (AAPH, >100% haemolysis) highlight their ability to protect erythrocyte integrity (Figure 10b). The activity was concentration-dependent, comparable in some cases to the effect of ascorbic acid (*p* > 0.05 for P1 at 5 mg/mL), and even significantly lower than ascorbic acid (*p* < 0.01 for P3–P5 at 20 mg/mL and 10 mg/mL). Materials P3 and P4, which have a lower extract content, showed a significantly better antihaemolytic effect compared to the similar ethanol control (P6 and P7, *p* < 0.0001), and haemolysis increases with decreasing material concentration. In the case of material P5, which contains a higher concentration of extract, the opposite trend is observed, meaning that the lower the concentration of the material, the better the antihaemolytic effect. At concentrations of 10 mg/mL and 5 mg/mL of different materials, the differences between P5 vs. P8 are significantly larger (*p* < 0.0001). These data support the potential of the materials as antioxidant and biocompatible agents, relevant for biomedical applications where oxidative stress plays a critical role, including in tissue healing processes.

## 3. Discussion

*P. nigrum* contains a rich spectrum of phenolic compounds and alkaloids with antioxidant, anti-inflammatory, and antimicrobial activity [46,130,131,132]. These secondary metabolites have numerous therapeutic properties, including promoting re-epithelialisation and wound closure [133]. According to literature data, the extract of *P. nigrum* highlights the presence of molecules with diverse roles, ranging from protection against oxidative stress to controlling bacterial colonisation [46]. Their integration into collagen matrices offers a promising strategy for developing functionalised materials with topical applications.

The polyphenol-rich profile, of which non-flavonoid compounds (16.13%), such as phenolic acids, tannins, alkaloids, and anthocyanins, were predominant [134]. In *P. nigrum*, UHPLC/MS-MS frequently reports gallic, caffeic, ferulic, synaptic acids and their derivatives, along with piperine [135]. A 2022 study highlighted the presence of compounds similar to those in our study, but with different values, with naringenin having a higher concentration compared to the other flavonoids. For the first time, phlorizin, phloretin, isorhamnetin, and abietic acid, compounds with strong antimicrobial, antioxidant, and re-epithelialization effects, were identified in the *P. nigrum* extract [41,42,136,137,138].

In human keratinocytes (HaCaT), protocatechuic acid protects against UVB by reducing intracellular ROS and MMP-1, demonstrating antioxidant photoprotective properties, and was also mentioned as an acetylcholinesterase inhibitor with a potential mechanism for modulating pruritus/cutaneous cholinergic signalling [127,130]. Another phenolic acid predominant in the *P. nigrum* seed extract was synapic acid, which has the ability to protect HaCaT keratinocytes from UVB by decreasing ROS and increasing DNA repair [36]. A formulation with 1% synaptic acid gel accelerated wound healing in diabetic rats by increasing collagen production and antioxidant capacity [36]. Abietic acid exhibits an anti-inflammatory effect (including topical, ear oedema models), promotes healing by increasing HUVEC angiogenesis, accelerates skin healing in mice [37,137,139,140], and has anti-atopic effects in a murine model of atopic dermatitis [141]. Microemulsion/emulgel formulations containing abietic acid have improved healing properties with an antioxidant role [137,142,143].

Among flavonoids, naringin exhibits properties that raise vascular permeability, promote angiogenesis, reduce ischaemia–reperfusion injury, and have been proposed for the survival of skin flaps. This compound is an adjuvant in the healing of poorly perfused wounds (flaps, ischaemic ulcers) [38]. Another flavonoid with a therapeutic topical role is isorhamnetin, which exhibits anti-UVB photoprotective properties by reducing ROS and DNA/protein/lipid damage and is anti-inflammatory for reactive skin/photoaging [138].

According to Sharma et al. [144], phlorizin has accelerated healing properties by increasing re-epithelialisation, angiogenesis, and reducing inflammation in burn models, as well as antibacterial/antioxidant properties [145]. Due to its antimicrobial properties, it has potential applications in contaminated chronic wounds. Phloretin has recently been incorporated into active dressings, demonstrating enhanced tissue regeneration in diabetic models due to its antioxidant, antimicrobial, and anti-inflammatory properties [46,133,134,135].

In the case of stilbenes, resveratrol was identified and quantified in the *P. nigrum* seed extract. Resveratrol exhibits antioxidant and anti-inflammatory properties relevant to skin conditions, as demonstrated by numerous studies [42,136]. In laboratory animals, it has been observed to lead to increased hydroxyproline secretion and increased tissue resistance (surgical healing). Additionally, resveratrol improves skin healing, scarring and reduces photoaging (it reduces UVB pigmentation in a preclinical model) [42].

The use of bioinformatics approaches provides a powerful framework for dissecting the individual contributions of phytochemicals within complex mixtures [146]. In the present study, the extract of *P. nigrum*, which is known to be rich in phenolic acids, stilbenes, anthocyanins, and flavonoids, was analysed using structure-based predictive techniques. By leveraging computational tools, it is possible to infer with high accuracy the pharmacological potential of these compounds based on their chemical structures [147]. The results suggest that the antimicrobial and anti-inflammatory properties of the extract may be linked to the interaction of its constituents with molecular targets such as monoamine oxidase A (MAO-A) and carbonic anhydrases. These findings support the hypothesis that in silico characterisation of natural compounds could guide the identification of key bioactive molecules responsible for therapeutic effects while also providing mechanistic insights into their biological activity.

Antioxidant activity was assessed using three techniques to figure out the potential mechanism of action for a combination of phytocompounds extracted from *P. nigrum* seeds. Phytochemical mixtures can be more active due to synergistic effects and may have a much lower cytotoxic potential [148,149]. The DPPH radical is a scavenger in alcoholic solvents; antioxidants react mainly through electron transfer (SET), hydrogen atom transfer (HAT), or the SPLET mechanism (proton loss followed by electron transfer) [150,151]. The FRAP method quantifies the reducing power toward the Fe^3+^ complex to Fe^2+^ at pH = 3.6; it is an SET method and does not detect chelation or radical scavenging per se [152]. It tends to favour compounds capable of efficiently donating electrons under acidic conditions. The CUPRAC method measures the reduction of Cu^2+^ to Cu^+^ at physiological pH. It is still a SET method but capable of measuring antioxidant capacity for both hydrophilic and lipophilic matrices [152].

The IC_50_ value of 41.14 ± 4.33 µL/mL indicates moderate radical-scavenging activity for a crude extract. The DPPH assay may undervalue antioxidants that are poorly soluble or sterically hindered in accessing the radical, such as alkaloid-rich matrices (e.g., piperine), which typically exhibit a weaker response relative to SET-type assays [153,154]. While the FRAP (2.15 ± 0.02 mM ET/mL) and CUPRAC (1.04 ± 0.03 mM ET/mL) methods confirm good reducing power. A higher FRAP value suggests that under acidic conditions, the extract compounds efficiently donate electrons to Fe^3+^–TPTZ.

*P. nigrum* extract has moderate scavenging (DPPH) and solid reducing power (FRAP/CUPRAC). This indicates a predominantly SET (electron donation) mechanism supported by a matrix rich in phenolic acids and, to a lesser extent, flavonoids, a result that correlates with the specialised literature reported for *P. nigrum* [35,155]. In the *P. nigrum* extract, phenolic acids having a catechol nucleus (e.g., caffeic, protocatechuic) or guaiacyl nucleus (e.g., ferulic, synaptic) considerably contribute to antioxidant properties via electron transfer processes proven in FRAP and CUPRAC assays [156,157], their ability to stabilise phenoxy radicals being enhanced by the presence of ortho-dihydroxyl groups, which not only facilitate the reduction of transition metal ions but also allow for Fe^2+^/Fe^3+^ chelation, thus limiting Fenton reactions and the formation of hydroxyl radicals [3,152,153] which translates biologically into the protection of the extracellular matrix and support for the wound healing process by reducing local oxidative stress. Piperine showed a modest signal in DPPH [158], but it can contribute through the FRAP method, so in biological systems, it can activate Nrf2 and anti-inflammatory pathways, which becomes relevant for healing [159].

Polar extracts from *P. nigrum* seeds (ethanolic, methanolic, hydroalcoholic, etc.) are rich in alkaloid amides (e.g., piperine) and polyphenols. The spectrum and potency differ visibly between these categories, and methodological differences explain some of the reported variability [5,20,46,160,161,162]. In the case of Gram-positive bacteria, ethanolic extracts and piperine-enriched fractions frequently achieve MIC < 100 μg/mL against *S. aureus* [5]. According to Chatterjee et al. [163], *P. nigrum* essential oil had moderate activity, primarily by affecting the outer membrane. *E. coli* was a recurring model for demonstrating increased membrane permeability and morphological alterations [164]. Another possible mechanism of action is the inhibition of efflux pumps (NorA, *S. aureus*) by piperine, and especially by its synthetic analogues [164], which potentiates the activity of ciprofloxacin and reduces the emergence of resistance [165].

The ethanolic extract of *P. nigrum* seeds demonstrated predominantly bacteriostatic activity (MIC values between 250–62.5 µL/mL) and relevant antibiofilm effects (MBEC values between 125–31.25 µL/mL), with greater sensitivity against Gram-positive bacteria (including *MRSA*) and a strain-dependent response in the case of Gram-negative bacteria (*P. aeruginosa*, *K. pneumoniae*, *P. mirabilis*), especially those isolated from infected wounds. The fact that the IC_50_ and MIC values were lower than those of the solvent, and that the MBEC was sometimes below the MIC, is consistent with the anti-virulence mechanisms previously described for piperine and the phenolic compounds in pepper, such as efflux pump inhibition, quorum-sensing disruption, and increased membrane permeability [163,166]. Overall, the data supports the use of the extract as an adjuvant in controlling the bioburden and biofilms in chronic wounds, where Gram-negative bacteria and staphylococci frequently co-colonise, and the persistence of the microbial biofilm hinders healing [167,168].

Hydroalcoholic (ethanolic or methanolic) extracts (rich in piperine) have been shown to be more active against Gram-positive bacteria [3,15,163,164] with useful activity against some Gram-negative bacteria (*Salmonella* sp.), but weak against *P. aeruginosa* [169,170], a result that correlates with the data presented. In some series, isolated piperine was similar or superior to the extract, highlighting the role of amides [171]. Isolated and purified piperine [171] was not necessarily the main active principle of the cell, but it disarms the pathogen by inhibiting certain virulence and pathogenicity factors, and it enhances the effects of antibiotics by inhibiting efflux, ensuring a potentially promising effect that was later used as an adjuvant [40,172].

The solvent and extraction method (maceration vs. Soxhlet vs. purified fractions) yield distinct chemical profiles and very different MIC values; sometimes, the ethanolic macerate has the best activity–complexity profile [173,174,175,176,177,178].

The inclusion of *P. nigrum* extract in the collagen matrix preserves the triple-helix structure and, at moderate doses, enhances the material’s thermal stability while providing clinically relevant antibacterial and antibiofilm activity, representing a major advantage for topical biomedical applications.

FTIR spectra confirmed the preservation of the collagen triple helix and, dose-dependently, the incorporation of aromatic/phenolic compounds from the *P. nigrum* extract. Spectral signatures indicate non-covalent interactions (hydrogen bonds and hydrophobic contacts) between alkaloid amides/phenols and collagen, which anticipates thermal stabilisation at low-to-medium loadings and plasticisation at high loadings. TGA-DSC confirms that the temperature of the Endo I events increases at P3 and P4 (more coherent network) and decreases at P5 (plasticising effect), while the mass loss slightly increases with the extract dose but remains below that of the ethanol controls (P6–P8) in the high-temperature window, with a better carbonisation yield. These effects are consistent with the aromatic/polyphenolic content of the extract. SEM mechanistically correlates the fact that a uniform amorphous coating of collagen granules was observed in materials P3 and P4 (microstructure compatible with stabilisation), while agglomeration/coalescence appears in P5, consistent with plasticisation [179], likely due to the specific carbohydrate content of the extract (glycosylated phenols, carbohydrates). Overall, the inclusion of *P. nigrum* extract does not compromise the thermal stability of the composites; at moderate doses, it increases the initial denaturation temperature, while at high doses, it only induces slight plasticisation without changing the degradation pathway (dehydration, denaturation, pyrolysis, carbon oxidation), the differences being quantitative, not qualitative.

The uniform distribution within the functionalised materials P3 and P4 favours diffusion and promotes the release of active principles, thus increasing compatibility with better synergy with KZ at low–medium doses. For P5 powder, the formation of agglomerates leads to the appearance of areas with uneven distribution and weaker diffusion pathways, aspects that can justify the decrease in synergy.

The connection between the TPC profile and the FRAP/CUPRAC/TEAC data indicates that the primary antioxidant mechanism was electron-donating, which was compatible with the presence of phenolic compounds and aromatic amides in *P. nigrum*. Complete solubilisation in PBS eliminates extraction artefacts and allows attribution of the extract’s impact.

The *P. nigrum* extract, having a predominantly bacteriostatic profile, was evaluated for its effect when combined with a standard antibiotic. Cefazolin (KZ, a first-generation cephalosporin) is frequently used in the management of chronic wounds dominated by Gram-positive bacteria due to its tissue penetration and safety profile [180]. However, the spectrum of this antibiotic against Gram-negative bacteria is limited (outer membrane barrier, β-lactamase production) [181,182]. In this context, cefazolin was used as a control antibiotic to assess whether the *P. nigrum* extract could enhance its activity against both Gram-positive and selected Gram-negative bacteria with intrinsic resistance. In the adapted diffusion test (synergy criterion Δ > 2 mm between MF + KZ and KZ), maximum synergy frequently occured at low to medium extract concentrations (P3 and P4), while at the high dose (P5) the effect no longer increases or even becomes modest—suggestive of diffusion limitations in agar, aggregation/chelation, or other matrix–antibiotic interactions at high concentrations [183,184]. For Gram-positive bacteria such as *S. aureus* ATCC and *S. epidermidis* ATCC, a robust synergy effect was observed, whereas the response against *MRSA* remained indifferent, and antagonism was even detected in *E. faecalis* (consistent with the intrinsic resistance of enterococci to cephalosporins) [185]. Among Gram-negative bacterial strains, a variable and strain-dependent effect was observed. No changes were detected for *P. aeruginosa* ATCC or *K. pneumoniae* ATCC 13368, whereas synergy was observed for *E. coli* (more pronounced in the ATCC strain than in the clinical isolate), *P. mirabilis* (ATCC and clinical isolate), and *P. aeruginosa* 1014 (wound isolate). Synergy at low to medium doses was consistent with possible adjuvant actions of the extract—such as inhibition of NorA-type efflux pumps in staphylococci, increased membrane permeability, and anti-quorum-sensing or anti-biofilm effects—which may reduce the effective dose of cefazolin. At high doses, suboptimal mixing, aggregation of hydrophobic compounds, or physicochemical interactions with cefazolin could attenuate the observed enhancement [186]. In line with previous reports [186,187,188,189], where staphylococci and Gram-negative bacilli (*P. aeruginosa*, *Proteus*, *E. coli*, *Klebsiella*) frequently co-colonise, these data indicated that materials functionalised with *P. nigrum* can enhance the activity of KZ against *S. aureus* and *S. epidermidis*, as well as a subset of Gram-negatives (e.g., *E. coli*, *P. mirabilis*, *P. aeruginosa* 1014), but not universally (e.g., *P. aeruginosa* ATCC, *K. pneumoniae* ATCC).

For topical applications, the extract loading was optimised, and materials P3 and P4 led to the maximisation of synergy and diffusion. The combination has potential as an adjuvant for bioburden control in wounds colonised with Gram-positive bacteria and certain Gram-negative bacteria.

Quantitatively, by estimating the FICI, the results indicate that variant P3 (low dose of extract) enhances the effectiveness of KZ against some bacteria relevant to infected wounds, especially those isolated from infected wounds. The largely additive beneficial effects were particularly evident against *S. aureus* strains (including *MRSA*) and Gram-negative bacteria such as *E. coli* and *P. mirabilis*, where the combinations reduced the antibiotic dose needed to inhibit growth and eradicate the biofilm. In contrast, in the case of *E. faecalis* and *S. epidermidis*, collagen powders had limited or even antagonistic effects, suggesting that their applicability is pathogen-selective. High doses of the extract (P5) or the solvent (P6–P8) generated indifference or antagonism, highlighting the importance of optimising the concentrations of active principles in the support base (collagen–arginine). Overall, cefazolin–P3/P4 combinations may represent a promising strategy for controlling polymicrobial infections in chronic wounds, excluding *E. faecalis* and *S. epidermidis*, with the potential to reduce bacterial resistance and antibiotic requirements. In general, *S. epidermidis* is a bacterial strain which is normally found in the skin microbiome; it can only become pathogenic under certain conditions [190]. The observed synergy with KZ may be related to the ability of the main phenolic acids and flavonoids (naringin, isorhamnetin, 3,4-dihydroxybenzoic acid, and sinapic acids) specific to the *P. nigrum* extract to disrupt bacterial membranes and inhibit biofilm formation, thus improving the antibiotic’s penetration and efficacy, especially against Gram-negative bacteria.

According to literature data, both the extract and piperine can exhibit in vitro cytotoxicity at concentrations of a few tens of μg/mL [4,191,192,193]. Therefore, the interpretation of antimicrobial activity should consider selectivity towards host cells and the target application (topical, pharmaceutical). For topical use, biocompatibility was assessed on a cell line suitable for these applications (HaCaT cell line). The resulting materials demonstrated good biocompatibility (viability over 85%) and haemocompatibility (haemolysis less than 1%), suggesting compliance with the acceptability criteria specified for Class 3 and 2b medical devices [194,195].

In a skin wound, excessive ROS prolong the inflammatory phase and degrades the ECM through matrix metalloproteinases (MMPs). Conversely, an increased reducing antioxidant capacity (SET-type mechanisms) decreases oxidative stress and preserves pro-repair factors. The elevated FRAP and CUPRAC responses observed indicate strong redox capacity, supporting the potential use of the system as an adjuvant in regenerative applications. This is consistent with HPLC confirmation of phenolic acids and flavonoids, such as caffeic, ferulic, sinapic, naringin, isorhamnetin, phlorizin, and phloretin [196,197,198,199], compounds known to accelerate healing. Their incorporation into a collagen matrix, therefore, represents a promising pharmaceutical form.

Recent studies have reported that piperine formulated in a topical hydrogel accelerates wound healing, leading to faster closure and improved histological outcomes [47,200,201]. These findings indicate that the bioactive compounds in black pepper can support the re-epithelialisation, angiogenesis, and collagen deposition. Additional studies on topical *P. nigrum* extracts in excision models have shown superior healing compared with certain plant-based controls, suggesting that the polyphenolic matrix and piperine may act synergistically, exerting moderate antioxidant, anti-inflammatory, and antimicrobial effects [46,47,202].

Collagen can interact with membrane proteins and phospholipids, providing mechanical and electrostatic stabilisation, along with arginine residues, which can sequester metal ions (Fe^2+^/Cu^2+^) and reduce Fenton reactions [203,204]. Arginine, a precursor of NO, can additionally contribute to redox cytoprotectants. Collagen also functions as a release matrix for polyphenolic antioxidants. *P. nigrum* extract provides phenolic compounds capable of SET (confirmed by FRAP/CUPRAC), scavenging peroxyl radicals, and, for catechol reasons, chelating Fe, thus limiting AAPH-induced lipid peroxidation [4,205,206]. Material functionalisation with extract (P3–P5) significantly reduced haemolysis in the AAPH model, an effect directly attributed to the phenolic compounds in black pepper [207], while collagen–arginine samples with equivalent solvent volumes (P6–P8) generally showed even less haemolysis, suggesting an additive/synergistic effect between the collagen barrier, metal chelation (Col/Arg), and phenolic antioxidants. The dose dependence (20 > 10 > 5 mg/mL) indicated a role for total antioxidant load and material–cell interactions. The controls validate the model (AAPH was the high haemolysis control, and ascorbic acid was the antihemolytic control). Overall, P3–P5 provide protection through the antioxidants in *P. nigrum*, while P6–P8 maximise hemocompatibility/antihemolysis through the synergy between collagen–arginine and the extract, offering the best protective profile at the maximum tested dose.

Materials with *P. nigrum* extract (P3–P5) significantly reduced AAPH-induced haemolysis, and collagen–arginine formulations (P6–P8) exhibited the strongest antihemolytic activity, suggesting a synergistic effect between the phenolic antioxidants and the stabilisation of erythrocyte membranes conferred by the protein matrix.

## 4. Materials and Methods

### 4.1. Plant Collection and Extract Preparation

Black pepper seeds (*P. nigrum* L.) were bought from a verified local supplier and processed into a fine powder with a laboratory mill (Retsch ZM 200, Haan, Germany). To perform ultrasound-assisted extraction (USAE), 5 g of material was suspended in 25 mL of 50% ethanol (*v*/*v*) and ultrasounded (frequency 40 kHz) at room temperature for 15 min in a laboratory ultrasonic bath (Daihan, WUC-D10H, Gangwon-do, Republic of Korea). The plant residue was kept in the flask (cycle 1) after the mixture was filtered with filter paper of 17–30 μm pore size (Fisher Scientific, Waltham, MA, USA). The residue was re-extracted twice with 25 mL of 50% ethanol under the same UAE conditions (cycles 2 and 3). The water in the ultrasonic bath was cooled to 24 °C between cycles. After mixing the three filtrates, 60 mL of extract was obtained. To prevent degradation of the thermolabile phytocompounds, all extractions were performed at room temperature. Insoluble particles (plant fragments, polysaccharides, and proteins) were removed from the three extracts (cycles 1, 2, and 3) by centrifugation for ten minutes at 8000 rpm and 4 °C to decrease turbidity. Before analysis, the supernatant was kept at −20 °C. Prior to antimicrobial testing, the extract underwent sterile filtering (0.22 μm PES).

### 4.2. HPLC-MS/MS Analysis

The quantitative determination of phenolic compounds in the extract was carried out using an UltiMate 3000 UHPLC system coupled to a Q-Exactive Focus Hybrid Quadrupole-Orbitrap mass spectrometer equipped with a heated electrospray ionisation (HESI) source (Thermo Scientific, Bremen, Germany), following the conditions described by Onache et al. (2023) [205]. Chromatographic separation was achieved on a Kinetex C18 column (100 × 2.1 mm, 1.7 µm; Phenomenex, Torrance, CA, USA) using a binary solvent system composed of (A) water containing 0.1% formic acid and (B) methanol containing 0.1% formic acid. Additionally, UHPLC–MS/MS screening was performed to identify the specific phenolic constituents present in *Piper nigrum* extract. Data acquisition was conducted in full scan and negative ionisation mode over an *m*/*z* range of 120–1800, with a resolving power of 70,000 FWHM at *m*/*z* 200. Variable data-independent acquisition (vDIA) MS^2^ analysis was performed at a resolution of 35,000, using isolation windows and scan ranges set as follows: 120–305 *m*/*z*, 195–405 *m*/*z*, 295–505 *m*/*z*, 395–1000 *m*/*z*, and 1000–1800 *m*/*z*. High-resolution mass spectrometry (HRMS) data were processed using Compound Discoverer software (version 2.1), employing an untargeted metabolomics workflow. Compound identification was supported through accurate mass matching against several reference databases, including ChemSpider (www.chemspider.com), PubChem (https://pubchem.ncbi.nlm.nih.gov), MassBank (https://massbank.eu/MassBank/), and SpectraBase (www.spectrabase.com) (access date between 1–5 September 2025).

### 4.3. Bioactive Polyphenolic Characterisation

Total Phenolic Content (TPC). With a few small adjustments, TPC was determined using the methodology described by Corbu et al. [208]. In short, 180 µL of water, 20 µL of Folin–Ciocalteu reagent, 200 µL of 7% Na_2_CO_3_, 100 µL of water, and 20 µL of sample or gallic acid solutions were combined. The resulting solutions were centrifuged for 10 min at 8000 rpm after being incubated for 50 min at room temperature in the dark. The absorbance at 765 nm was then measured after 200 µL of each combination was put onto a 96-well microplate. For concentrations between 25 and 250 μg/mL of gallic acid, a calibration curve was created (R^2^ = 0.9965). The amount of phenolic content in the extract was expressed as µg gallic acid equivalent (GAE)/mL.

Total Flavonoids Content (TFC). The determination of TFC was carried out according to the aluminium chloride method [208]. In brief, 0.2 mL of sample/standard, 0.2 mL of 10% sodium acetate, and 0.2 mL of 2.5% AlCl_3_ solution were mixed with 0.2 mL of 50% ethanol. After stirring, these solutions were kept at room temperature for 35 min in the dark before centrifugation at 8000 rpm. The absorbance of the combination was measured at 430 nm. The calibration curve was performed for quercetin concentrations between 10 and 250 μg/mL (R^2^ = 0.9998).

DPPH assay. The antioxidant activity based on DPPH radical scavenging was evaluated using a method adapted from Marinas et al. [175]. The procedure involved mixing 300 µL of 0.3 mM DPPH methanolic solution with 300 µL of the sample or Trolox standard solution (Sigma-Aldrich, Burlington, MA, USA). The solutions were incubated at room temperature for 30 min in the dark before being tested for absorbance at 517 nm. A calibration curve was developed using Trolox concentrations ranging from 5 to 80 µM (R^2^ = 0.9984). The results were expressed as IC_50_ values, reflecting the sample’s radical scavenging capacity (µL/mL).

The CUPRAC method is based on the reduction of a cupric complex, neocuproine, by antioxidants in copper form. To reduce copper ions, 120 µL of sample or standard solution was combined with 100 µL of 10 mM CuCl_2_, 100 µL of 7.5 mM neocuproine, and 100 µL of 1 M ammonium acetate buffer (pH 7.0), as previously described [175]. After gentle mixing, the reaction mixtures were kept in the dark for 20 min before centrifugation at 8000 rpm for 10 min. After transferring 200 µL of each combination into a 96-well microplate, the absorbance was measured at 450 nm. The calibration curve was generated using Trolox solutions with concentrations ranging from 0.25 to 1.25 mM (R^2^ = 0.9997). The antioxidant capacity was reported as µmol Trolox equivalents per millilitre of extract (TE/mL).

FRAP assay. The determination of the antioxidant capacity of iron reduction was performed by the FRAP assay method [175]. The FRAP reagent was freshly made by combining acetate buffer (300 mM, pH 3.6), TPTZ solution (10 mM in 40 mM HCl), and FeCl_3_·6H_2_O solution (20 mM) in a volume ratio of 10:1:1. It was then warmed to 37 °C before use. To perform the test, 200 µL of each reaction mixture was pipetted onto 96-well microplates. After incubation, the absorbance was measured at 593 nm. The calibration curve was created with Trolox standards ranging from 10 to 250 µM (R^2^ = 0.9993). The results were represented in µmol Trolox equivalents (TE/mL extract).

TEAC assay. The Trolox Equivalent Antioxidant Capacity (TEAC) test was carried out using the technique described by Re et al. [209], with slight modifications. After combining 7 mM ABTS with 2.45 mM potassium persulfate, the reaction mixture was left to stand at room temperature for 12–16 h in the dark to create a stable ABTS^+^ radical cation stock solution. The stock was diluted with ethanol to achieve an absorbance of around 0.70 at 734 nm, which yielded the workable ABTS^+^ solution. The test involved mixing 360 µL of the ABTS^+^ working solution with 40 µL of the sample or Trolox standard solution. The solutions were incubated in the dark for 20 min before centrifugation at 8000 rpm for 10 min. Next, 200 µL of each supernatant was placed into a 96-well microplate, and the absorbance was measured. The calibration curve was constructed using Trolox concentrations between 20 and 200 µM (R^2^ = 0.9967). The radical scavenging capacity was expressed as Trolox equivalents (µmol TE/mL extract).

### 4.4. Bioinformatics Methods

The compounds identified from *Piper nigrum* extract were retrieved from the PubChem database, from which the SMILES files were obtained. Physicochemical properties were subsequently evaluated, with a particular focus on compliance with Lipinski’s Rule of Five (MW < 500, LogP < 5, number of hydrogen bond donors ≤ 5, and number of hydrogen bond acceptors ≤ 10), using the SwissADME platform. To further assess pharmacokinetic behaviour, the DeepPK platform was employed to predict absorption, distribution, metabolism, excretion, and toxicity (ADMET) profiles, yielding a comprehensive set of descriptors. Finally, the SMILES files were submitted to the SwissTargetPrediction tool to identify potential molecular targets for the selected compounds.

### 4.5. Antimicrobial Activity of P. nigrum Extract

#### 4.5.1. Microbial Strains

Reference microbial strains (*Staphylococcus aureus* ATCC 25923, MRSA 43300, *S. epidermidis* ATCC 12228, *E. faecalis* ATCC 15021, *Pseudomonas aeruginosa* ATCC 27853, *Escherichia coli* ATCC 25922, *K. pneumoniae* ATCC 13368, *P. mirabilis* ATCC 29245) and clinical isolates [210] from infected wounds (*S. aureus* sc pl, *E. coli* C10E, *P. aeruginosa* 1014, *K. pneumoniae* B1K, *P. mirabilis* 11P) were used to evaluate the antimicrobial activity. The microbial strains used are part of the strain collection of the Faculty of Biology, University of Bucharest.

#### 4.5.2. Quantitative Evaluation of Antimicrobial Activity

The quantitative evaluation of antimicrobial activity was conducted using the serial two-fold dilution method in liquid medium (Tryptone Soy Broth) within 96-well microplates. The tested concentration range was between 500 and 0.16 µL/mL. In parallel, serial dilutions of 50% ethanol were prepared under identical conditions to serve as negative controls. Each well was inoculated with 10 µL of a microbial suspension standardised to 1.5 × 10^8^ CFU/mL, obtained from 18–24 h cultures. Each well had a final volume of 110 µL. Blank samples for each concentration were produced in the same experimental setup. The microplates were then incubated at 37 °C for 24 ± 2 h. The minimum inhibitory concentration (MIC) was measured visually as the lowest concentration that showed no apparent microbial growth and spectrophotometrically by measuring absorbance at 620 nm with a FlexStation 3 UV-Vis spectrophotometer. The MIC values were determined as the lowest extract concentration reducing microbial viability by more than 10% of the untreated control. As these values represent inhibitory thresholds, data are reported as reproducible single concentrations. The minimum microbicidal concentration (MMC) was calculated by plating 5 µL from each well that showed no growth on solid agar medium and incubating it for 20–24 h at 37 °C. The lowest concentration at which no colony development occurred was designated as the MMC.

#### 4.5.3. Microbial Adherence of *P. nigrum* Extract

Following a quantitative assessment of antibacterial activity, the slime test was used to measure microbial adherence. After incubation, the wells were fixed with 120 µL of methanol and allowed to dry, then stained with 120 µL of 0.1% crystal violet solution. The dyed biomass was solubilised in 120 µL of 33% CH_3_COOH, and the absorbance of the resultant solution was measured at 490 nm to quantify cell adherence. MBEC values represent the minimum concentration at which microbial adherence was reduced by more than 90% compared to the untreated control strain. The MBEC assay was performed in triplicate.

### 4.6. Collagen—Plant Extract Formulation

The collagen hydrogel used in this study was prepared according to Maier et al. [68] and had the characteristics described by Zorilă et al. [69]. Subsequently, within this study, the matrix was functionalised according to the procedures detailed below. In short, the collagen solution (Type I, bovine origin) obtained earlier was dialysed until a pH greater than 5.5 was reached. Prior to use, the solution was thawed from −20 °C and slowly equilibrated to room temperature. Collagen (10 g) was mixed with L-arginine (2.24 g, Sigma-Aldrich, Burlington, MA, USA, ≥98% purity) and homogenised by ultra-turraxing with milling balls using an IKA ULTRA-TURRAX^®^ Tube Drive (IKA—Werke GmbH, Staufen, Germany), equipped with a stainless-steel milling ball microtubule (Ø: 5 mm). Homogenisation was performed at speed 9 for 180 s, with 30 s pauses between cycles to keep the temperature below 25 °C. The procedure generated a uniform paste, free of visible particles, which was immediately used for the subsequent functionalisation steps (P2). To observe the structural changes, a control sample (P1) was obtained by homogenising the collagen hydrogel without L-arginine under the same conditions. For functionalisation, 2 mL (P3), 4 mL (P4), and 8 mL (P5) of *P. nigrum* extract were added to this composite, followed by homogenisation at speed 9 for 30 s. Control samples similar to P3, P4, and P5 were prepared, in which the extract was replaced with 50% ethanol. All variants were freeze-dried and ground to obtain fine powders.

### 4.7. Physico-Chemical Characterisation

*ATR-FTIR.* Fourier-transform infrared spectra of the collagen-based composites (P1–P8) were obtained using a Cary 630 FTIR spectrometer equipped with an ATR (attenuated total reflectance) accessory and controlled by Agilent MicroLab FTIR v5.5.1989 software (Agilent Technologies, Inc., Santa Clara, CA, USA). Spectral data were collected in the range of 4000–650 cm^−1^, with a total of 400 scans per sample and a resolution of 4 cm^−1^.

SEM. The surface morphology of the samples was examined using a Nova NanoSEM 630 scanning electron microscope (FEI Company, Hillsboro, OR, USA) operated at an accelerating voltage of 10 kV. Prior to imaging, all samples were sputter-coated with a thin layer of gold (Au) to improve electrical conductivity and image quality.

DLS analysis. The hydrodynamic particle size was determined using a Beckman Coulter Delsa™ Nano C instrument (Beckman Coulter, Brea, CA, USA) based on dynamic light scattering (DLS). During the measurement, particles were illuminated by dual 30 mW laser diodes, generating time-dependent fluctuations in the scattered light intensity. The scattered light was collected at a fixed angle of 165° for particle size determination and detected using a highly sensitive photodetector. All measurements were carried out at room temperature, and each sample was analysed in triplicate. Data processing was performed using Delsa™ Nano software version 3.73. Prior to analysis, the dispersions were ultrasonicated for 10 min to ensure proper homogenisation.

TGA-DSC analysis. Thermogravimetric and differential scanning calorimetry (TG–DSC) analyses of the samples were carried out using a Netzsch STA 449C Jupiter thermal analyser (NETZSCH-Gerätebau GmbH, Selb, Germany). The samples were placed in open alumina crucibles and heated from room temperature to 900 °C at a constant rate of 10 K·min^−1^ under a continuous flow of dried air (50 mL·min^−1^). An empty alumina crucible was used as the reference.

### 4.8. Phytochemical Composition and Antioxidant Activity

Total phenolic content (TPC) and total flavonoid content (TFC). These parameters were evaluated using the methods described earlier (Section 4.3). For each material, stock solutions of 10 mg/mL were prepared in PBS (pH 7.4). Total phenolic content was expressed as mg gallic acid equivalent (GAE)/g sample, and total flavonoid content (TFC) was expressed as mg quercetin equivalent (QE)/g sample.

Antioxidant activity. The DPPH, FRAP, CUPRAC and TEAC assays were evaluated using the methods described previously (Section 4.3). For each material, stock solutions of 10 mg/mL were done in PBS (pH 7.4). To compare the methods and identify the main antioxidant mechanisms, all results were expressed as mM TE (Trolox Equivalent)/g material.

### 4.9. Antimicrobial Activity of Collagen Composite

#### 4.9.1. Qualitative and Quantitative Evaluation of Antimicrobial Activity

The behaviour of the materials developed in the presence of a currently used antibiotic in clinical practice (cefazolin, KZ) was evaluated both qualitatively and quantitatively. Stock solutions were prepared with final concentrations of collagen materials (9.1 mg/mL) and cefazolin (0.9 mg/mL) in PBS (pH 7.4). To test the quality of antimicrobial activity, microbial suspensions were prepared, adjusted to 1.5 × 10^8^ CFU/mL (0.5 McFarland standard), from 18–24 h cultures grown. Antimicrobial activity was determined by an adapted diffusion method [98]. A volume of 10 µL of each sample was distributed onto the inoculated medium. The inhibition area around the spot level was measured in mm. The synergistic effect was considered when the difference between the diameter of the antibiotic’s inhibition zone (for 0.9 mg/mL) and that of the mixture (collagen material and KZ) was greater than 2 mm. If the difference falls between the interval 2–0, the effect was considered indifferent, while negative values were attributed to the antagonistic effect.

The antimicrobial activity was quantitatively assessed using the serial two-fold microdilution method in 96-well microplates. The tested concentration range was 500 to 0.009 mg/mL, prepared in Tryptone Soy Broth (TSB; American Biorganics, Buffalo, NY, USA). Each well was inoculated with a standardised microbial suspension equivalent to 0.5 McFarland, prepared in phosphate-buffered saline (PBS) from the tested strains. The procedure followed the same protocol as described in Section 4.5.2. Plates were incubated at 37 °C for 24 h, and the results were interpreted by calculating the fractional inhibitory concentration index (FICI) using the relationship [211]:

$$\mathrm{FICI}\;=\;\frac{MIC\;KZ\;comb}{MIC\;KZ\;alone}+\frac{MIC\;Col\;material\;comb}{MIC\;Col\;material\;alone}$$

The FICI values were interpreted as follows: FICI ≤ 0.5 indicated synergism; 0.5 < FICI ≤ 1 was considered an additive effect; 1 < FICI ≤ 2 an indifference effect; and FICI > 2 an antagonistic effect. Each determination was performed in triplicate for accuracy of results.

#### 4.9.2. Microbial Adherence

After completing the quantitative antimicrobial analysis, microbial adherence was assessed using the slime assay method according to Section 4.5.3.

### 4.10. Biocompatibility Assessment

In order to assess the biocompatibility of the developed materials, human keratinocytes (HaCaT cell line) were cultured in Dulbecco’s Modified Eagle Medium (DMEM, Sigma-Aldrich, St. Louis, MO, USA) supplemented with 10% foetal bovine serum (FBS, Gibco, Grand Island, NY, USA) at 37 °C in a humidified atmosphere with 5% CO_2_. Keratinocytes were seeded at a density of 3 × 10^4^ cells/cm^2^ in 96-well plates and allowed to adhere overnight, after which the growth medium was changed with a fresh one containing 500 µg/mL of each sample. After 24 h, the cells were processed according to cell viability tests.

MTT Assay. This test was used to assess cell viability and proliferation in the presence of the tested samples and is based on the internalisation of the MTT [3-(4,5-dimethylthiazol-2-yl)-2,5-diphenyltetrazolium] compound into living cells and its reduction to purple formazan crystals by the mitochondria of metabolically active cells. Briefly, the cells were incubated for 2 h with 1 mg/mL MTT solution at 37 °C, 95% humidity and 5% CO_2_. The formed formazan crystals were dissolved in isopropanol, and the absorbance was measured at 595 nm on a FlexStation 3 plate reader (Molecular Devices, San Jose, CA, USA).

Lactate Dehydrogenase (LDH) Release Assay. The culture medium was collected after 24 h of exposure to the samples, and LDH release was measured using the Cytotoxicity Detection Kit PLUS (Roche, NY, USA) according to the manufacturer’s instructions. An equal volume (50 µL) of culture supernatant and a prepared catalyst-dye reaction mixture was combined and incubated for 30 min under dark conditions. To terminate the reaction, 50 µL of a stopping solution was added. Absorbance readings were then taken at 490 nm using a microplate reader. (Flex Station 3, Molecular Devices, San Jose, CA, USA).

Wound Healing Assay. To evaluate the impact of the tested suspensions on wound healing, cells were seeded at a density of 10^5^ cells/cm^2^ in 24-well plates to form confluent monolayers. After 24 h, linear scratches were introduced using a sterile 200 μL pipette tip, followed by a medium wash to eliminate residual cell fragments. Images of the wound areas were captured at multiple time points (0, 6 and 24 h) post-treatment, continuing until complete closure of the cell-free region was observed for at least one of the treatment conditions. Cell migration was visualised and captured using an Olympus IX73 microscope (Olympus, Tokyo, Japan). The percentage of the wound area was quantified using ImageJ software (version 1.54g), and wound closure was calculated with the formula: wound closure (%) = [(Gap T0 − Gap TΔ)/Gap T0] × 100%, where T0 is the area (%) of the wound measured right after the scratch procedure and TΔ is the area (%) of the wound measured after each time point assessed (6 and 24 h). For statistical evaluation, 16 images were analysed per treatment condition.

### 4.11. Blood Contact Behaviour

The haemolytic activity was evaluated using ram red blood cells (RBCs), following the procedure described by Geana et al. [51], with slight modifications. To avoid clotting, 9 mL of blood was combined with 1 mL of a 10% citric acid-dextrose solution. After centrifuging the mixture for 10 min at 4 °C at 5000 rpm, the plasma fraction was carefully extracted. The resulting RBC pellet was resuspended in PBS (0.1 M, pH 7.4) after three rounds of washing. To conduct the experiment, 100 µL of each test sample (20 mg/mL) was mixed with 0.4 mL of RBC suspension and incubated at 37 °C for 60 min. Following incubation, the resulting solutions were centrifuged at 5000 rpm for 10 min at 4 °C, and the absorbance of the supernatants was measured at 540 nm with a 96-well microplate reader. Phosphate-buffered saline served as the negative control, while 1% Triton X-100 was used as the positive control.

The collagen powders’ anti-haemolytic properties were assessed using a previously described spectrophotometric technique [51], with minor changes. For every sample, a 20 mg/mL stock solution was prepared in PBS (working concentrations were 20, 10, and 5 mg/mL materials in PBS, pH = 7.4). The sample solution (100 µL) was combined with 400 µL of 10% erythrocyte suspension. After 20 min of incubation at 37 °C, 200 µL of AAPH (2,2′-Azobis (2-amidinopropane) dihydrochloride) (final concentration 0.2 M) was added to evaluate its effects. To provide enough advanced oxidation and allow for a clear assessment of the tested compound’s capacity to protect against AAPH-induced oxidative stress, the mixes were incubated for 4 h at 37 °C. After centrifuging the samples for 10 min at 5000 rpm and 4 °C, the supernatant absorbance was measured at 540 nm. The relative haemolysis was determined by comparing it to the haemolysis in the AAPH-treated negative control, which was defined as 100% total haemolysis. PBS without AAPH therapy was used as the positive control. Inhibitory activity was represented as a percentage of haemolysis inhibition, and each set of experiments was carried out in triplicate.

### 4.12. Statistical Analysis

All data has been provided as mean values ± standard deviation (SD) from duplicate/triplicate experiments. GraphPad Prism version 10.0 was used to conduct statistical analysis. Two-way ANOVA was used to compare IC_50_ values and anti-haemolytic activities. Tukey’s multiple comparison test with a single pooled variance was then applied. The antioxidant activity, TPC, TFC, and hemo-/biocompatibility data were examined using one-way ANOVA, followed by Tukey’s post hoc test with a single pooled variance. Statistical significance was established at *p* < 0.05.

## 5. Conclusions

Polyphenols, including phenolic acids, stilbenes, anthocyanins, and flavonoids, which provide a potent antioxidant capacity through electron transfer mechanisms (FRAP, CUPRAC), were abundant in the hydroalcoholic extract of *P. nigrum* seeds. FTIR and SEM analyses confirmed the integration of phenolic compounds into the collagen–arginine matrix without protein denaturation, with the formation of a uniform amorphous film at low and medium doses and the appearance of agglomerates at high doses. TGA–DSC data showed thermal stabilisation at moderate extract concentrations and a plasticising effect at high concentrations. The extract exhibited predominantly bacteriostatic antibacterial activity and antibiofilm effects, with clear synergy with cefazolin, especially at medium doses (P4). The synergy with KZ is due to phenolic acids and flavonoids found in *P. nigrum* extract, which break bacterial membranes and hinder biofilm formation, increasing the antibiotic’s potency against Gram-negative bacteria. Tests on keratinocytes (HaCaT) confirmed biocompatibility, and haemolytic evaluations demonstrated an excellent safety profile, with protection against AAPH-induced oxidative stress. Overall, collagen powders with *P. nigrum* extract at moderate concentrations (P4) combine stability, antimicrobial activity, and cellular compatibility, making them a promising adjuvant for the topical treatment of chronically infected wounds.

## Figures and Tables

**Table 1:** Analytical parameters of antioxidant activity and phenolic composition of *P. nigrum* extract. The results are expressed as mean ± SD (n = 3).

Analytical Parameter	*P. nigrum* Extract Used for Formulation	*P. nigrum* Dry Extract (4.06 mg/mL Extract)
TPC	497.57 ± 4.81 µg GAE/mL	122.6 ± 1.19 mg GAE/g
TFC	80.25 ± 1.40 µg QE/mL	19.8 ± 0.35 mg QE/g
DPPH (IC_50_)	41.14 ± 4.33 µL/mL	167.03 ± 17.58 µg/mL
CUPRAC	1.04 ± 0.03 mM ET/mL	256.16 ± 7.39 µM ET/g
FRAP	2.15 ± 0.02 mM ET/mL	529.56 ± 4.93 µM ET/g
TEAC	0.75 ± 0.04 mM ET/mL	185.76 ± 9.77 µM ET/g

**Table 2:** Identification and quantitative data regarding the bioactive compounds in the *P. nigrum* hydroalcoholic extract (n = 2).

Compound Name	R.T. (min)	Accurate Mass [M − H]^+^/[M − H]^−^	MS^2^ Fragments (*m*/*z*)	Concentration (µg/L)
Gallic acid	1.97	169.0133	169.0133; 125.0231	nd
3,4-Dihydroxybenzoic acid	3.98	153.0183	109.0281	2107.18 ± 81.97
2,5-Dihydroxybenzoic acid	6.58	53.0183	109.0342; 153.0261	33.32 ± 0.54
4-Hydroxybenzoic acid	6.28	137.0232	118.9650; 96.9588; 71.0124	nd
Caffeic acid	7.98	179.0342	135.0440	96.97 ± 7.40
Syringic acid	8.18	197.0446	182.0212; 166.9976; 153.0547; 138.0311; 123.0075	nd
Vanillic acid	3.48	167.0341	152.0116; 108.0229; 123.0441	98.75 ± 5.32
p-Coumaric acid	8.67	163.0388	119.0489	nd
Sinapic acid	8.77	223.0608	79.7560; 95.9510; 118.9651	2365.64 ± 96.99
Ferulic acid	8.87	193.0499	178.0262; 134.0361	nd
Ellagic acid	9.71	300.9992	300.9990	43.25 ± 3.84
Abscisic acid	9.95	263.1290	179.9803; 191.9454	nd
Abietic acid	21.80	303.1996	96.9587; 183.0113; 79.9559	343.01 ± 19.62
**Σ phenolic acids (µg/L)**				**5088.11**
Catechin	7.53	289.0718	109.0282; 123.0349; 125.0232; 137.0232; 151.0390; 203.0708	15.48 ± 1.02
Epicatechin	8.13			44.38 ± 2.93
Myricetin *	8.61	319.0443	178.9986; 164.9263; 151.0036; 137.0244; 107.0125	nd
Rutin	9.42	609.1457	301.0352; 300.0276	nd
Naringin	9.18	579.1714	177; 151	498.81 ± 35.02
Hesperidin	9.36	609.1825	377.0876	nd
Taxifolin	8.62	303.0488	147.0440; 257.0814	13.01 ± 0.58
Isorhamnetin	9.36	317.0665	300.0277	253.61 ± 12.68
Kaempferol	11.94	285.0384	151.0389; 117.0180	9.19 ± 0.64
Apigenin	11.97	269.0451	117.0333; 151.0027; 107.0126	25.38 ± 1.11
Pinocembrin	12.76	255.0657	213.0551; 151.0026; 107.0125	nd
Chrysin	13.81	253.0500	143.0491; 145.0284; 107.0125; 209.0603; 63.0226, 65.0019	14.67 ± 0.42
Galangin	14.01	269.0451	169.0650; 143.0491	8.59 ± 0.46
Vitexin	8.97	431.0986	341.0664; 269.0454; 240.0422; 197.0606	85.93 ± 4.83
**Σ flavonoids (µg/L)**				**969.05**
Phlorizin	9.47	319.0443	107.0553	170.87 ± 10.17
Phloretin	10.90	609.1457	93.0332; 121.0283	189.21 ± 17.71
**Σ dihydrochalcones (µg/L)**				**360.08**
Resveratrol	9.52	227.0710	185.0813, 143.0337	412.78 ± 27.99
Isorapontigenin	9.87	257.0816	241.0504; 125.0231; 175.0392; 217.0502; 175.0393	nd
**Σ other compounds (µg/L)**				**412.78**
Piperine *	14.03	286.1436	285.0999; 201.0996; 173.0408	-
Piperolactam A	12.54	264.0666	166.9095; 83.0489; 69.0332	-
Piperolactam D	13.52	294.0772	85.0281	-
Cepharadione A	1.98	304.0616	210.0379	-
Tetracosanoic acid	23.66	367.3582	367.3583; 281.2488	-
3,4-Methylenedioxycinnamic acid	9.95	191.0341	191.0343; 147.0443; 119.0492	-
2-Butenedioic acid	0.73	115.0037	115.0024; 71.0124	-

nd—Not detected; * [M − H]^+^—not quantified.

**Table 3:** Quantitative assessment of antimicrobial activity expressed by minimum inhibitory concentration (MIC), minimum microbicidal concentration (MMC) and minimum biofilm eradication concentration (MBEC), respectively. The analysis was done in triplicate (n = 3).

Strains	MIC (µL/mL)		MMC (µL/mL)		MBEC (µL/mL)	
	*P. nigrum* Extract	EtOH	*P. nigrum* Extract	EtOH	*P. nigrum* Extract	EtOH
*S. aureus* ATCC 25923	125	250	>500	>500	62.5	250
MRSA 43300	125	500	>500	>500	31.25	500
*S. aureus* sc pl	125	500	>500	>500	125	500
*S. epidermidis* ATCC 12228	125	500	500	500	250	500
*E. faecalis* ATCC 15021	250	>500	>500	>500	125	>500
*E. coli* ATCC 25922	125	250	500	500	125	250
*E. coli* C10E	500	250	>500	>500	125	250
*P. aeruginosa* ATCC 27853	62.5	250	250	250	62.5	62.5
*P. aeruginosa* 1014	125	500	500	500	62.5	500
*K. pneumoniae* ATCC 13368	125	125	500	>500	125	125
*K. pneumoniae* B1K	62.5	125	>500	>500	62.5	125
*P. mirabilis* ATCC 2924S	125	250	>500	>500	125	250
*P. mirabilis* 11P	250	250	>500	>500	62.5	250

**Table 4:** Nomenclature compounds, chemical ID extracted from PubChem (https://pubchem.ncbi.nlm.nih.gov/docs/about, accessed on 23 September 2025) and smiles files were presented in Table 5.

Compound	Class of Compounds	Pub Chem ID	Molecular Weight (g/mol)	SMILES
Piperine	alkaloids—amide	638024	285.136	C1CCN(CC1)C(=O)/C=C/C=C/C2=CC3=C(C=C2)OCO3
Piperolactam A	alcaloid tetraciclic	3081016	265.074	COC1=C(C2=C3C(=C1)C(=O)NC3=CC4=CC=CC=C42)O
Piperolactam D	alcaloid	14039008	295.084	COC1=C2C3=C(C4=CC=CC=C4C=C3NC2=O)C(=C1OC)O
Cepharadione A	isoquinoline alkaloid	94577	305.069	CN1C2=CC3=CC=CC=C3C4=C2C(=CC5=C4OCO5)C(=O)C1=O
Tetracosanoic acid	long-chain saturated fatty acids	11197	368.365	CCCCCCCCCCCCCCCCCCCCCCCC(=O)O
3,4-Methylenedioxycinnamic acid	acid cinamic	643181	192.042	C1OC2=C(O1)C=C(C=C2)/C=C/C(=O)O
2-Butenedioic acid	dicarboxylic acid	723	116.011	C(=CC(=O)O)C(=O)O
Piperonylic acid	aromatic carboxylic acid	7196	166.027	C1OC2=C(O1)C=C(C=C2)C(=O)O

**Table 5:** Physico-chemical characterisation of natural compounds using SwissADME platform [62].

Molecule	Rotatable Bonds	H-Bond Acceptors	H-Bond Donors	TPSA (Å^2^)	LogP	Bioavailability Score
Piperine	4	3	0	38.77	3.38	0.55
Piperolactam A	1	3	2	62.32	2.14	0.55
Piperolactam D	2	4	2	71.55	2.33	0.55
Cepharadione A	0	4	0	57.53	2.26	0.55
Tetracosanoic acid	22	2	1	37.3	5.62	0.85
3,4-Methylenedioxycinnamic acid	2	4	1	55.76	1.8	0.85
2-Butenedioic acid	2	4	2	74.6	0.32	0.85
piperonylic acid	1	4	1	55.76	1.44	0.85

**Table 6:** ADME character of natural compounds predicted using Deep-PK tool.

Compound Name	Human Intestinal Absorption	Skin Permeability	Blood–Brain Barrier	CYP 1A2 Inhibitor	CYP 1A2_Substrate	CYP 2C19 Inhibitor	CYP 2C19_Substrate	CYP 2C9 Inhibitor	CYP 2C9 Substrate	CYP 2D6 Inhibitor	CYP 2D6 Substrate	CYP 3A4 Inhibitor	CYP 3A4 Substrate	Half-Life of Drug
Piperine	0.99	−2.08	1	yes	no	no	no	no	no	yes	yes	yes	no	0.25
Piperolactam A	0.99	−1.3	0.83	yes	yes	yes	no	no	yes	no	no	no	no	0.20
Piperolactam D	0.98	−1.85	0.28	yes	yes	yes	no	no	no	no	no	no	no	0.26
Cepharadione A	0.99	−1.9	0.99	yes	yes	yes	no	yes	yes	yes	no	yes	no	0.17
Tetracosanoic acid	0.82	−3.05	0.94	no	no	yes	no	no	no	no	no	no	no	0.54
3,4-Methylenedioxycinamic acid	0.99	−2.74	0.98	no	no	no	no	no	yes	no	no	no	no	0.36
2-Butenedioic acid	0.72	−3.21	0.77	no	no	no	no	no	no	no	no	no	no	0.79
Piperonylic acid	0.98	−3.28	0.92	no	no	no	no	no	no	no	no	no	no	0.36

**Table 7:** Toxicity profile of natural compounds extracted from *P. nigrum*.

Compound Name	AMES Mutagenesis	Carcinogenesis	Liver Injury I (DILI)	Liver Injury I (DILI)	Eye Corrosion	hERG Blockers
Piperine	0.001	0.273	Safe	0.352	0	0.118
Piperolactam A	0.968	0.466	Toxic	0.983	0	0.017
Piperolactam D	0.642	0.178	Toxic	0.97	0	0.022
Cepharadione A	0.999	0.293	Toxic	0.976	0	0
Tetracosanoic acid	0	0.136	Toxic	0.651	0.694	0.566
3,4-Methylenedioxycinamic acid	0.003	0.309	Toxic	0.508	0.021	0.004
2-Butenedioic acid	0	0.033	Toxic	0.502	0.988	0.065
Piperonylic acid	0.005	0.26	Toxic	0.532	0.192	0.242

**Table 8:** Molecular targets obtained by running the SwissTargetPrediction bioinformatics tool.

Compound Name	Target	Probability
Piperine	Monoamin oxidase B	1.00
Piperolactam A	Cyclin-dependent kinase 2	0.24
Tetracosanoic acid	Fatty acid binding protein adipocyte	0.37

**Table 9:** Thermogravimetric and calorimetric analysis of samples P1–P8: mass loss (%) and thermal effects (ºC).

Sample	Mass Loss (%)				Thermal Effect (°C)			
	RT-210 °C	210–280 °C	280–470 °C	470–700 °C	Endo I	Endo II	Endo III	Exo I
P1	2.83	16.06	47.70	36.18	73.1	218.6	255.7	559.2
P2	1.26	15.27	49.94	35.20	73.9	224.4	258.0	565.8
P3	2.99	15.89	47.89	33.80	82.8	220.9	258.1	569.8
P4	3.11	14.91	48.98	33.81	82.1	220.5	255.8	555.0
P5	1.46	15.49	50.85	34.15	68.0	220.0	254.6	571.5
P6	1.18	15.93	51.84	34.67	77.0	222.6	260.8	578.2
P7	1.65	15.60	50.21	34.38	83.8	224.2	258.2	580.2
P8	1.05	15.46	51.98	34.16	68.0	223.1	258.1	569.3

**Table 10:** Zone of inhibition (mm) in the diffusion test for cefazolin (KZ, 0.91 mg/mL), functionalized/unfunctionalized collagen material (MF, mg/mL), and the MF + KZ combination on reference bacterial strains and clinical isolates. Interaction classification: synergy (s., Δ ≤ 2 mm), indifference (indif., 2 < Δ < 0 mm), and antagonism (ant., Δ < 0 mm). The analysis was done in triplicate (n = 3).

Strains	Code	MF	MF + KZ	KZ	Diff (Δ, Interp.)
*S. aureus* ATCC 25923	P3	0.00 ± 0.00	35.00 ± 1.41	31.00 ± 1.41	4 (s.)
	P4	0.00 ± 0.00	35.00 ± 1.41		4 (s.)
	P5	0.00 ± 0.00	35.00 ± 1.41		4 (s.)
	P6	0.00 ± 0.00	33.00 ± 1.41		2 (s.)
	P7	0.00 ± 0.00	33.00 ± 1.41		2 (s.)
	P8	0.00 ± 0.00	33.00 ± 1.41		2 (s.)
MRSA 43300	P3	0.00 ± 0.00	21.00 ± 0.00	20.00 ± 0.00	1 (indif.)
	P4	0.00 ± 0.00	21.50 ± 0.71		1.5 (indif.)
	P5	0.00 ± 0.00	21.00 ± 0.00		1 (indif.)
	P6	0.00 ± 0.00	20.00 ± 0.00		0 (indif.)
	P7	0.00 ± 0.00	19.50 ± 0.71		−0.5 (ant.)
	P8	0.00 ± 0.00	21.50 ± 0.71		1.5 (indif.)
*S. aureus* sc pl	P3	0.00 ± 0.00	30.00 ± 0.00	28.00 ± 0.00	2 (s.)
	P4	0.00 ± 0.00	29.00 ± 0.00		1 (indif.)
	P5	0.00 ± 0.00	28.50 ± 0.71		0.5 (indif.)
	P6	0.00 ± 0.00	22.50 ± 0.71		−5.5 (ant.)
	P7	0.00 ± 0.00	28.50 ± 0.71		0.5 (indif.)
	P8	0.00 ± 0.00	28.00 ± 0.00		0 (indif.)
*E. faecalis* ATCC 15021	P3	0.00 ± 0.00	0.00 ± 0.00	20.00 ± 0.00	−20 (ant.)
	P4	0.00 ± 0.00	0.00 ± 0.00		−20 (ant.)
	P5	0.00 ± 0.00	0.00 ± 0.00		−20 (ant.)
	P6	0.00 ± 0.00	0.00 ± 0.00		−20 (ant.)
	P7	0.00 ± 0.00	0.00 ± 0.00		−20 (ant.)
	P8	0.00 ± 0.00	20.00 ± 1.42		0 (indif.)
*S. epidermidis* ATCC 12228	P3	15.00 ± 1.41	35.00 ± 1.42	31.00 ± 1.41	4 (s.)
	P4	0.00 ± 0.00	35.00 ± 1.42		4 (s.)
	P5	0.00 ± 0.00	34.00 ± 0.00		3 (s.)
	P6	0.00 ± 0.00	32.00 ± 1.42		2 (s.)
	P7	0.00 ± 0.00	33.00 ± 2.83		1 (indif.)
	P8	0.00 ± 0.00	33.00 ± 1.42		2 (s.)
*E. coli* ATCC 25922	P3	0.00 ± 0.00	20.50 ± 0.71	18.00 ± 0.00	2.5 (s.)
	P4	0.00 ± 0.00	21.00 ± 0.00		3 (s.)
	P5	0.00 ± 0.00	21.00 ± 0.00		3 (s.)
	P6	0.00 ± 0.00	19.00 ± 0.00		1 (indif.)
	P7	0.00 ± 0.00	19.00 ± 0.00		1 (indif.)
	P8	0.00 ± 0.00	19.00 ± 0.00		1 (indif.)
*E. coli* C10E	P3	0.00 ± 0.00	15.50 ± 0.71	13.50 ± 0.71	2 (s.)
	P4	0.00 ± 0.00	16.00 ± 0.00		2.5 (s.)
	P5	0.00 ± 0.00	15.00 ± 0.00		1.5 (indif.)
	P6	0.00 ± 0.00	15.00 ± 0.00		1.5 (indif.)
	P7	0.00 ± 0.00	15.00 ± 0.00		1.5 (indif.)
	P8	0.00 ± 0.00	14.50 ± 0.71		1 (indif.)
*P. aeruginosa* ATCC 27853	P3	0.00 ± 0.00	0.00 ± 0.00	0.00 ± 0.00	-
	P4	0.00 ± 0.00	0.00 ± 0.00		-
	P5	0.00 ± 0.00	0.00 ± 0.00		-
	P6	0.00 ± 0.00	0.00 ± 0.00		-
	P7	0.00 ± 0.00	0.00 ± 0.00		-
	P8	0.00 ± 0.00	0.00 ± 0.00		-
*P. aeruginosa* 1014	P3	0.00 ± 0.00	15.50 ± 0.71	12.50 ± 0.71	3 (s.)
	P4	0.00 ± 0.00	16.00 ± 0.00		3.5 (s.)
	P5	0.00 ± 0.00	14.00 ± 1.42		1.5 (indif.)
	P6	0.00 ± 0.00	14.50 ± 0.71		2 (s.)
	P7	0.00 ± 0.00	14.50 ± 0.71		2 (s.)
	P8	0.00 ± 0.00	14.50 ± 0.71		2 (s.)
*K. pneumoniae* ATCC 13368	P3	0.00 ± 0.00	0.00 ± 0.00	0.00 ± 0.00	-
	P4	0.00 ± 0.00	0.00 ± 0.00		-
	P5	0.00 ± 0.00	0.00 ± 0.00		-
	P6	0.00 ± 0.00	0.00 ± 0.00		-
	P7	0.00 ± 0.00	0.00 ± 0.00		-
	P8	0.00 ± 0.00	0.00 ± 0.00		-
*K. pneumoniae* B1K	P3	0.00 ± 0.00	19.00 ± 0.00	16.00 ± 0.00	3 (s.)
	P4	0.00 ± 0.00	18.00 ± 0.00		2 (s.)
	P5	0.00 ± 0.00	17.50 ± 0.71		1.5 (indif.)
	P6	0.00 ± 0.00	18.00 ± 0.00		2 (s.)
	P7	0.00 ± 0.00	17.50 ± 0.71		1.5 (indif.)
	P8	0.00 ± 0.00	18.00 ± 0.00		2 (s.)
*P. mirabilis* ATCC 292 4S	P3	0.00 ± 0.00	24.00 ± 0.00	20.50 ± 0.71	3.5 (s.)
	P4	0.00 ± 0.00	23.00 ± 0.00		2.5 (s.)
	P5	0.00 ± 0.00	23.50 ± 0.71		3 (s.)
	P6	0.00 ± 0.00	22.50 ± 0.71		2 (s.)
	P7	0.00 ± 0.00	22.50 ± 0.71		2 (s.)
	P8	0.00 ± 0.00	22.50 ± 0.71		2 (s.)
*P. mirabilis* 11P	P3	0.00 ± 0.00	24.50 ± 0.71	22.00 ± 0.00	2.5 (s.)
	P4	0.00 ± 0.00	24.50 ± 0.71		2.5 (s.)
	P5	0.00 ± 0.00	24.00 ± 0.00		2 (s.)
	P6	0.00 ± 0.00	23.50 ± 0.71		1.5 (indif.)
	P7	0.00 ± 0.00	23.50 ± 0.71		1.5 (indif.)
	P8	0.00 ± 0.00	24.00 ± 0.00		2 (s.)

**Table 11:** Evaluation of the behaviour of the mixture of the two components, including the fractional minimum inhibitory concentration of cefazolin and functional materials (10 mg of the mixture comprises 0.91 mg of cefazolin and 9.09 mg of functionalised material). The analysis was done in triplicate (n = 3).

Strains	Sample	MIC (µg/mL) Bioactive Composite	MIC (µg/mL) Bioactive Composite	FICa	MIC (µg/mL) Antibiotic	MIC (µg/mL) Combination	FICb	FICI (Interp.)
*S. aureus* ATCC 25923	P3	781.25	1.7754	0.0023	0.3125	0.1777	0.56875	0.5710 (Ad.)
	P4	781.25	1.7754	0.0023	0.3125	0.1777	0.56875	0.5710 (Ad.)
	P5	1562.5	3.5508	0.0023	0.3125	0.3555	1.1375	1.1398 (Indif)
	P6	3125	3.5508	0.0011	0.3125	0.3555	1.1375	1.1386 (Indif)
	P7	3125	3.5508	0.0011	0.3125	0.3555	1.1375	1.1386 (Indif)
	P8	6250	3.5508	0.0006	0.3125	0.3555	1.1375	1.1381 (Indif)
MRSA 43300	P3	1953.13	568.1300	0.2909	113.7500	56.8750	0.5	0.7909 (Ad.)
	P4	1953.13	568.1300	0.2909	113.7500	56.8750	0.5	0.7909 (Ad.)
	P5	976.56	1136.2500	1.1635	113.7500	113.7500	1	2.1635 (Antag.)
	P6	1953.13	568.1300	0.2909	113.7500	56.8750	0.5	0.7909 (Ad.)
	P7	1953.13	568.1300	0.2909	113.7500	56.8750	0.5	0.7909 (Ad.)
	P8	3906.25	1136.2500	0.2909	113.7500	113.7500	1	1.2909 (Indif)
*S. aureus* sc pl	P3	1562.5	3.5508	0.0023	0.6250	0.3555	0.5688	0.571 (Ad.)
	P4	3125	7.1016	0.0023	0.6250	0.7109	1.1375	1.1398 (Indif)
	P5	1562.5	7.1016	0.0045	0.6250	0.7109	1.1375	1.1420 (Indif)
	P6	50,000	14.2031	0.0003	0.6250	1.4219	2.275	2.2753 (Antag.)
	P7	50,000	14.2031	0.0003	0.6250	1.4219	2.275	2.2753 (Antag.)
	P8	50,000	14.2031	0.0003	0.6250	1.4219	2.275	2.2753 (Antag.)
*S. epidermidis* ATCC 12228	P3	781.25	1.7754	0.0023	0.1563	0.1777	1.1375	1.1398 (Indif)
	P4	781.25	3.5508	0.0045	0.1563	0.3555	2.275	2.2795 (Antag.)
	P5	195.31	3.5508	0.0182	0.1563	0.3555	2.275	2.2932 (Antag.)
	P6	3125	3.5508	0.0011	0.1563	0.3555	2.275	2.2761 (Antag.)
	P7	1562.5	3.5508	0.0023	0.1563	0.3555	2.275	2.2772 (Antag.)
	P8	1562.5	3.5508	0.0023	0.1563	0.3555	2.275	2.2772 (Antag.)
*E. faecalis* ATCC 15021	P3	781.25	227.2500	0.2909	12.5000	22.7500	1.82	2.1109 (Antag.)
	P4	781.25	227.2500	0.2909	12.5000	22.7500	1.82	2.1109 (Antag.)
	P5	195.31	227.2500	1.1635	12.5000	22.7500	1.82	2.9835 (Antag.)
	P6	3125	454.5000	0.1454	12.5000	45.5000	3.64	3.7854 (Antag.)
	P7	1562.5	454.5000	0.2909	12.5000	45.5000	3.64	3.9309 (Antag.)
	P8	1562.5	909.0000	0.5818	12.5000	91.0000	7.28	7.8618 (Antag.)
*E. coli* ATCC 25922	P3	62,500	35.5100	0.0006	7.1100	3.5500	0.4993	0.4999 (Syn.)
	P4	62,500	35.5100	0.0006	7.1100	3.5500	0.4993	0.4999 (Syn.)
	P5	250,000	35.5100	0.0001	7.1100	3.5500	0.4993	0.4994 (Syn.)
	P6	250,000	35.5100	0.0001	7.1100	3.5500	0.4993	0.4994 (Syn.)
	P7	250,000	35.5100	0.0001	7.1100	3.5500	0.4993	0.4994 (Syn.)
	P8	125,000	35.5100	0.0003	7.1100	3.5500	0.4993	0.4996 (Syn.)
*E. coli* C10E	P3	625	284.0625	0.4545	56.8800	28.4400	0.5	0.9545 (Ad.)
	P4	625	284.0625	0.4545	56.8800	28.4400	0.5	0.9545 (Ad.)
	P5	625	284.0625	0.4545	56.8800	28.4400	0.5	0.9545 (Ad.)
	P6	62500	568.1250	0.0091	56.8800	56.8800	1	1.0091 (Indif.)
	P7	62500	284.0625	0.0045	56.8800	28.4400	0.5	0.5045 (Ad.)
	P8	3906.25	284.0625	0.0727	56.8800	28.4400	0.5	0.5727 (Ad.)
*P. aeruginosa* 1014	P3	488.28	142.0313	0.2909	56.8750	14.2188	0.25	0.5409 (Ad.)
	P4	1953.13	284.0625	0.1454	56.8750	28.4375	0.5	0.6454 (Ad.)
	P5	1953.13	142.0313	0.0727	56.8750	14.2188	0.25	0.3227 (Syn.)
	P6	976.56	284.0625	0.2909	56.8750	28.4375	0.5	0.7909 (Ad.)
	P7	1953.13	284.0625	0.1454	56.8750	28.4375	0.5	0.6454 (Ad.)
	P8	3906.25	142.0313	0.0364	56.8750	14.2188	0.25	0.2864 (Syn.)
*K. pneumoniae* B1K	P3	250,000	35.5078	0.0001	7.1094	3.5547	0.5	0.5001 (Ad.)
	P4	62,500	35.5078	0.0006	7.1094	3.5547	0.5	0.5006 (Ad.)
	P5	7812.50	35.5078	0.0045	7.1094	3.5547	0.5	0.5045 (Ad.)
	P6	250,000	35.5078	0.0001	7.1094	3.5547	0.5	0.5001 (Ad.)
	P7	250,000	35.5078	0.0001	7.1094	3.5547	0.5	0.5001 (Ad.)
	P8	250,000	35.5078	0.0001	7.1094	3.5547	0.5	0.5001 (Ad.)
*P. mirabilis* 292 4S ATCC	P3	62,500	71.0156	0.0011	14.2188	7.1094	0.5	0.5011 (Ad.)
	P4	62,500	71.0156	0.0011	14.2188	7.1094	0.5	0.5011 (Ad.)
	P5	250,000	71.0156	0.0003	14.2188	7.1094	0.5	0.5003 (Ad.)
	P6	125,000	142.0313	0.0011	14.2188	14.2188	1	1.0011 (Indif.)
	P7	125,000	142.0313	0.0011	14.2188	14.2188	1	1.0011 (Indif.)
	P8	250,000	142.0313	0.0006	14.2188	14.2188	1	1.0006 (Indif.)
*P. mirabilis* 11P	P3	250,000	142.0313	0.0006	28.4375	14.2188	0.5	0.5006 (Ad.)
	P4	62,500	142.0313	0.0023	28.4375	14.2188	0.5	0.5022 (Ad.)
	P5	250,000	142.0313	0.0006	28.4375	14.2188	0.5	0.5006 (Ad.)
	P6	125,000	284.0625	0.0023	28.4375	28.4375	1	1.0023 (Indif.)
	P7	125,000	284.0625	0.0023	28.4375	28.4375	1	1.0023 (Indif.)
	P8	250,000	284.0625	0.0011	28.4375	28.4375	1	1.0011 (Indif.)

Note: Indif. = Indifferent; Ad. = Adherence; Syn. = Synergism; Antag. = Antagonism.

**Table 12:** MBEC values (µg/mL) for functional collagen material combinations with cefazolin (KZ): 10 mg of the mixture comprises 0.91 mg of cefazolin and 9.09 mg of functionalised material. The analysis was done in triplicate (n = 3).

Strains	P3		P4		P5		P6		P7		P8		KZ
	P3 + KZ	KZ	P4 + KZ	KZ	P5 + KZ	KZ	P6 + KZ	KZ	P7 + KZ	KZ	P8 + KZ	KZ	
*S. aureus* ATCC 25923	3.91	0.36	1.95	0.18	7.81	0.71	15.63	1.42	15.63	1.42	15.63	1.42	0.31
MRSA 43300	312.5	28.44	625	56.88	625	56.88	625	56.88	625	56.88	625	56.88	56.88
*S. aureus* sc pl	7.81	0.71	7.81	0.71	7.81	0.71	15.63	1.42	15.63	1.42	15.63	1.42	0.625
*E. faecalis* ATCC 15021	250	22.75	250	22.75	250	22.75	500	45.5	500	45.5	>500	>45.5	25
*S. epidermidis* ATCC 12228	1.95	0.18	3.91	0.36	3.91	0.36	7.81	0.71	7.81	0.71	7.81	0.71	0.16
*E. coli* ATCC 25922	7.81	0.71	15.63	1.42	31.25	2.85	62.5	5.69	62.5	5.69	62.5	5.69	25
*E. coli* C10E	3.91	0.36	7.81	0.71	3.91	0.36	31.26	2.85	7.81	0.71	31.25	2.85	12.5
*P. aeruginosa* 1014	78.13	7.11	156.25	14.22	78.13	7.11	312.5	28.44	312.5	28.44	312.5	28.44	12.5
*K. pneumoniae* B1K	39.06	3.56	78.13	7.11	39.06	3.56	156.25	14.22	156.25	14.22	156.25	14.22	7.11
*P. mirabilis* ATCC 292 4S	78.13	7.11	156.25	14.22	156.25	14.22	312.5	28.44	312.5	28.44	312.5	28.44	31.25
*P. mirabilis* 11P	156.25	14.22	156.25	14.22	156.25	14.22	312.5	28.44	312.5	28.44	312.5	28.44	125

## Data Availability

The original contributions presented in this study are included in the article. Further inquiries can be directed to the corresponding author.

## Supplementary Materials

The following supporting information can be downloaded at https://www.mdpi.com/article/10.3390/antibiotics14111166/s1. Figure S1: Dose–response curve of the DPPH radical scavenging activity of the *P. nigrum* extract; Figure S2: Concentration-dependent effect of *P. nigrum* hydroalcoholic extract and the corresponding 50% ethanol solvent on the viability of (a) *S. aureus* ATCC25923, (b) *MRSA*, (c) *E. faecalis* ATCC15021, (d) *S. epidermidis*, (e) *S. aureus* sc pl, (f) *E. coli* ATCC 25922, (g) *E. coli* C10E, (h) *P. aeruginosa* 27853 ATCC, (i) *P. aeruginosa* 1014, (j) *K. pneumoniae* B1K, (k) *K. pneumoniae* 13368 ATCC, (l) *P. mirabilis* 292 4S ATCC and (m) *P. mirabilis* 11. Data are expressed as mean ± SD (n = 3).
