# Supplementary material for: Piper nigrum Extract as an Adjuvant in a Collagen System for Infected Wound Healing: Therapeutic Synergy and Biocompatibility
Source: Antibiotics (Basel). 2025 Nov 17;14(11):1166. doi: 10.3390/antibiotics14111166 (PMC12649488; doi:10.3390/antibiotics14111166)
Supplement: Supplementary file 1 [file antibiotics-14-01166-s001.zip › antibiotics-3966506-supplementary.pdf]

SUPPLEMENTARY MATERIAL

Piper nigrum extract as an adjuvant in a collagen system for infected wound healing: therapeutic synergy and biocompatibility

Virgina Silviana Becherescu Barbu [1,2], Ioana Cristina Marinas [3,4*], Diana Madalina Gaboreanu[5], Ionela Cristina Voinea[6], Oana Brincoveanu[7], Elisabeta-Irina Geana[8], Ovidiu-Cristian Oprea[9,10,11], Adina Boldeiu[7], Andra Maria Paun[1], Catalina Mares[1], Marian Angheloiu[4], Alice-Ștefania Șerbănoiu[5], Speranta Avram[1]

1 Department of Anatomy, Animal Physiology and Biophysics, Faculty of Biology, University of Bucharest, 050095 Bucharest, Romania (V.S.B.B., A.M.P., C.M., S.A.);
2 Phanos Technology SRL; 235300, Corabia, Romania (V.S.B.B.);
3 The Research Institute of the University of Bucharest (ICUB), 050095 Bucharest, Romania (I.C.M.);
4 SC Deltarom SRL - Centre for Research and Innovative Services in Advanced Biotechnology, Sos. Bucuresti-Giurgiu 1-2, Giurgiu 087040, Romania (I.C.M., M.A.);
5 Department of Botany and Microbiology, University of Bucharest, Faculty of Biology, Splaiul Independentei 91-95, Bucharest, R-050095, Romania (M.G., A.S.S.)
6 Department of Biochemistry and Molecular Biology, Faculty of Biology, University of Bucharest, 91-95 Splaiul Independentei, 050095 Bucharest, Romania (I.C.V.)
7 National Institute for Research and Development in Microtechnologies (IMT-Bucharest), 077190 Bucharest, Romania (O.B., A.B.);
8 National Research & Development Institute for Cryogenic and Isotopic Technologies-Ramnicu Valcea Romania, Strada Uzinei, No. 4, 240050 Râmnicu Vâlcea, Romania (E.I.G.);
9 National Centre for Micro- and Nanomaterials, National University of Science and Technology Politehnica Bucharest, 060042 Bucharest, Romania (O.C.O.);
10 Academy of Romanian Scientists, 050045 Bucharest, Romania (O.C.O.);
11 Department of Inorganic Chemistry, Physical Chemistry and Electrochemistry, Faculty of Chemical Engineering and Biotechnologies, National University of Science and Technology Politehnica of Bucharest, 011061 Bucharest, Romania (O.C.O.)
* Correspondence: ioana-cristina.marinas@icub.unibuc.ro

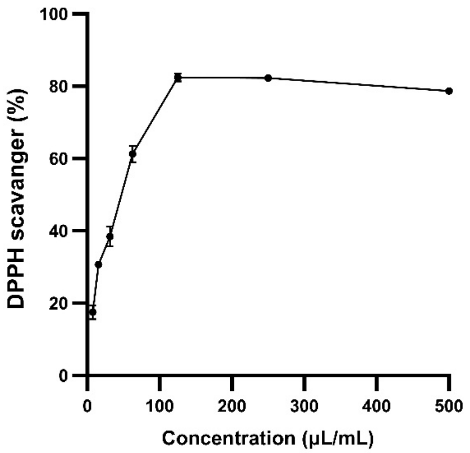

Figure S1. Dose–response curve of the DPPH radical scavenging activity of the *P. nigrum* extract

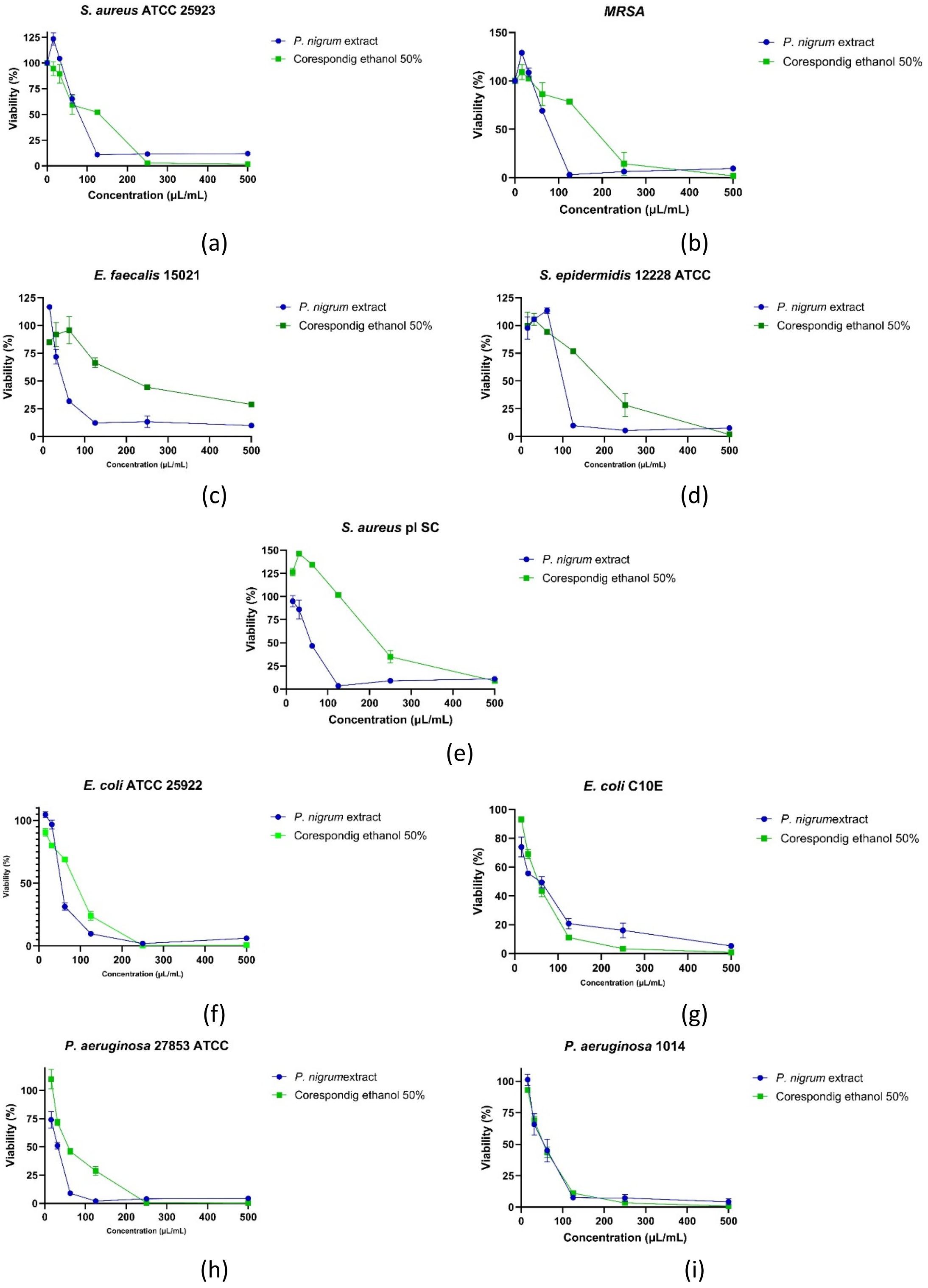
S. aureus ATCC 25923
P. nigrum extract
Corespondig ethanol 50%
Viability (%)
Concentration (µL/mL)
(a)
MRSA
P. nigrum extract
Corespondig ethanol 50%
Viability (%)
Concentration (µL/mL)
(b)
E. faecalis 15021
P. nigrum extract
Corespondig ethanol 50%
Viability (%)
Concentration (µL/mL)
(c)
S. epidermidis 12228 ATCC
P. nigrum extract
Corespondig ethanol 50%
Viability (%)
Concentration (µL/mL)
(d)
S. aureus pl SC
P. nigrum extract
Corespondig ethanol 50%
Viability (%)
Concentration (µL/mL)
(e)
E. coli ATCC 25922
P. nigrum extract
Corespondig ethanol 50%
Viability (%)
Concentration (µL/mL)
(f)
E. coli C10E
P. nigrumextract
Corespondig ethanol 50%
Viability (%)
Concentration (µL/mL)
(g)
P. aeruginosa 27853 ATCC
P. nigrumextract
Corespondig ethanol 50%
Viability (%)
Concentration (µL/mL)
(h)
P. aeruginosa 1014
P. nigrum extract
Corespondig ethanol 50%
Viability (%)
Concentration (µL/mL)
(i)

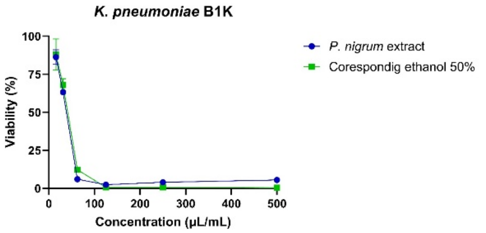

(j)

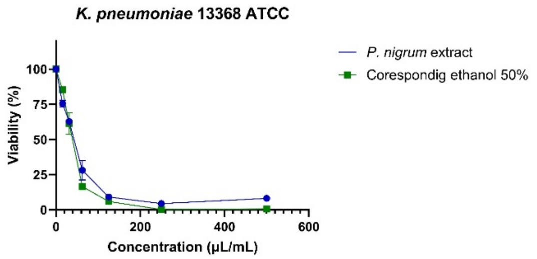

(k)

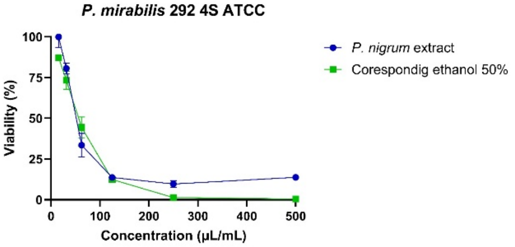

(l)

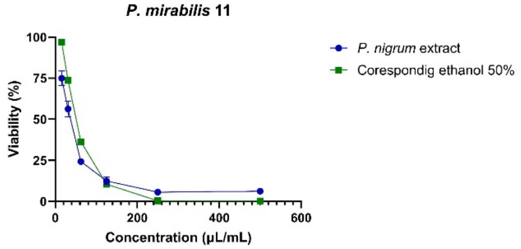

(m)

Figure S2. Concentration-dependent effect of *P. nigrum* hydroalcoholic extract and the corresponding 50% ethanol solvent on the viability of (a) *S. aureus* ATCC25923, (b) *MRSA, (c) E. faecalis* ATCC15021, (d) *S. epidermidis,* (e) *S. aureus* sc pl, (f) *E. coli ATCC 25922*, (g) *E. coli C10E*, (h) *P. aeruginosa 27853 ATCC*, (i) *P. aeruginosa 1014*, (j) *K. pneumoniae B1K*, (k) *K. pneumoniae* 13368 ATCC, (l) *P. mirabilis* 292 4S ATCC and (m) *P. mirabilis* 11. Data are expressed as mean ± SD (n = 3).
